# TCR and Inflammatory Signals Tune Human MAIT Cells to Exert Specific Tissue Repair and Effector Functions

**DOI:** 10.1016/j.celrep.2019.08.050

**Published:** 2019-09-17

**Authors:** Tianqi Leng, Hossain Delowar Akther, Carl-Philipp Hackstein, Kate Powell, Thomas King, Matthias Friedrich, Zoe Christoforidou, Sarah McCuaig, Mastura Neyazi, Carolina V. Arancibia-Cárcamo, Joachim Hagel, Fiona Powrie, Oxford IBD Investigators, Raphael Sanches Peres, Val Millar, Daniel Ebner, Rajesh Lamichhane, James Ussher, Timothy S.C. Hinks, Emanuele Marchi, Chris Willberg, Paul Klenerman

**Affiliations:** 1 Peter Medawar Building for Pathogen Research, South Parks Road, Oxford OX1 3SY, UK; 2 The Kennedy Institute of Rheumatology, Roosevelt Dr., Oxford OX3 7FY, UK; 3 Translational Gastroenterology Unit, Nuffield Department of Medicine, University of Oxford, Oxford OX3 9DU, UK; 4 Target Discovery Institute, Roosevelt Dr., Oxford OX3 7FZ, UK; 5 Department of Microbiology and Immunology, University of Otago, Otago, New Zealand; 6 NIHR Biomedical Research Centre, John Radcliffe Hospital, Oxford OX3 9DU, UK; 7 Respiratory Medicine Unit, Nuffield Department of Medicine Experimental Medicine, University of Oxford, Oxford OX3 9DU, UK; 8 Department of Microbiology and Immunology, Peter Doherty Institute for Infection and Immunity, University of Melbourne, Melbourne, VIC 3000, Australia

**Keywords:** MAIT cells, effector functions, TCR signaling, cytokines, tissue repair

## Abstract

MAIT cells are an unconventional T cell population that can be activated through both TCR-dependent and TCR-independent mechanisms. Here, we examined the impact of combinations of TCR-dependent and TCR-independent signals in human CD8^+^ MAIT cells. TCR-independent activation of these MAIT cells from blood and gut was maximized by extending the panel of cytokines to include TNF-superfamily member TL1A. RNA-seq experiments revealed that TCR-dependent and TCR-independent signals drive MAIT cells to exert overlapping and specific effector functions, affecting both host defense and tissue homeostasis. Although TCR triggering alone is insufficient to drive sustained activation, TCR-triggered MAIT cells showed specific enrichment of tissue-repair functions at the gene and protein levels and in *in vitro* assays. Altogether, these data indicate the blend of TCR-dependent and TCR-independent signaling to CD8^+^ MAIT cells may play a role in controlling the balance between healthy and pathological processes of tissue inflammation and repair.

## Highlights

• Activation of human MAIT cells is TCR-dependent or TCR-independent and enhanced by TL1A
• TCR-dependent and TCR-independent triggering induces distinct transcriptional responses
• TCR-dependent triggering of MAIT cells induces a tissue-repair program
• Data integration with *in vivo* studies in mice indicates a shared transcriptome

## Graphical Abstract

**Figure:**
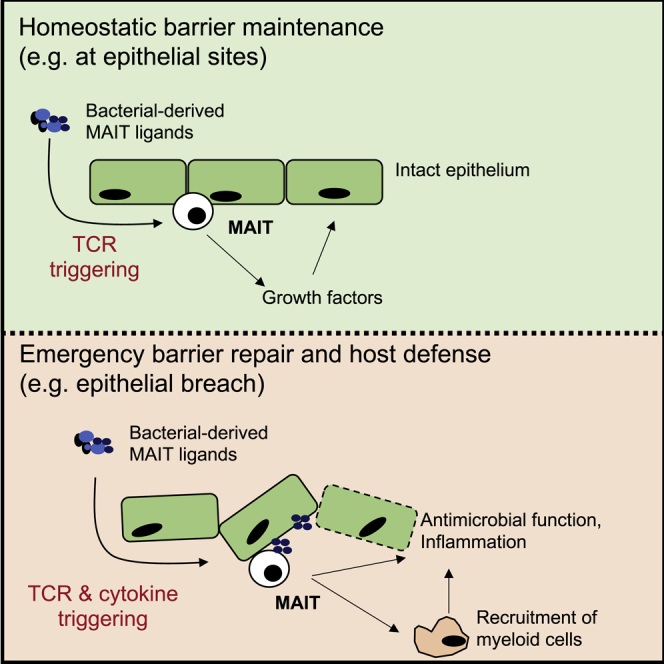

## Teaser

Leng et al. explore the consequences of activation of human MAIT cells via their TCR and/or cytokines, including the gut-associated TNF-superfamily member TL1A. TCR triggering reveals a transcriptional program linked to tissue-repair functions seen *in vivo*, consistent with a homeostatic role for these cells in epithelia.

## Introduction

Human innate and adaptive immune systems form a critical partnership in immune defense against microorganisms. Studies have revealed several types of unconventional T lymphocytes that sit at the bridge between innate and adaptive immunity, including mucosal-associated invariant T (MAIT) cells (Godfrey et al., 2015). MAIT cells are abundant in human blood and enriched most substantially in the liver (Dusseaux et al., 2011). They are marked by high surface expression of the C-type lectin molecule CD161, and they bear the semi-invariant T cell receptor (TCR) Vα7.2-Jα33/12/20, which restricts them to the evolutionary conserved, non-polymorphic major histocompatibility complex (MHC) class I-related protein 1 (MR1) (Kjer-Nielsen et al., 2012, Reantragoon et al., 2013). MAIT cells recognize microbially derived riboflavin synthesis intermediates presented by MR1 (López-Sagaseta et al., 2013, Ussher et al., 2014a). MR1 tetramers loaded with riboflavin and folate intermediates have been developed, enabling the specific detection and characterization of human and mouse MAIT cells (López-Sagaseta et al., 2013, Rahimpour et al., 2015, Reantragoon et al., 2013).

Despite this specific antigen recognition as an effector T cell, MR1-TCR signaling alone is insufficient to fully activate MAIT cells (Turtle et al., 2011). To achieve sufficient activation, TCR signaling is supported by other costimulatory signals, such as CD28, and by cytokines, such as interleukin (IL)-18 and IL-12 (Ussher et al., 2014b). This is true in mouse cells examined *in vivo*. Normal expansion is only seen if ligand is delivered with a toll-like receptor (TLR) stimulus (Chen et al., 2017).

This behavior has prompted investigation into the responsiveness of MAIT cells to innate signals, including IL-12, IL-18, IL-15, and type I interferons (IFNs) (Sattler et al., 2015, Ussher et al., 2014b, van Wilgenburg et al., 2016). *In vitro* studies in human cells have shown that cytokines such as IL-12 and IL-18 can, in combination, activate MAIT cells in a fully TCR-independent manner (Ussher et al., 2014b). Cytokine-stimulated CD161^2+^CD8^+^ T cells, including MAIT cells, may exert effector functions by secretion of cytokines and upregulation of granzyme (Gr) B (Billerbeck et al., 2010, Kurioka et al., 2015). We and others have highlighted a role for MAIT cells in viral infections, in which MAIT cell activation was TCR independent but depended on IL-18 in synergy with IL-12, IL-15, and/or the type I interferons IFN-α/β (Loh et al., 2016, van Wilgenburg et al., 2016), with a critical protective role *in vivo* (Wilgenburg et al., 2018). Thus, it is clear that MAIT cells can be activated via TCR-dependent and TCR-independent pathways. However, the diversity of functions triggered by different cytokines compared with those triggered by TCR signaling has yet to be defined.

The specific functions of MAIT cells elicited by cytokines are particularly relevant in mucosal tissues, such as the gut, where local signaling may be critical in defining the balance between host defense responses and tolerance. Data on IL-17-expressing skin-homing mouse CD8^+^ T cells, an innate-like T cell population that mirrors some critical features of MAIT cells, indicate that they display a tissue-repair phenotype rather than a pure inflammatory phenotype in response to TCR triggering via commensal-associated ligands (formyl peptides restricted by H2M3) (Linehan et al., 2018). The authors propose that responses to commensals driven by TCR could support a role for such T cells in tissue homeostasis. This behavior may extend to more broadly include innate-like T cells restricted by MHC1b molecules, which are evolutionarily ancient (Klenerman and Ogg, 2018).

Tumor necrosis factor (TNF)-like protein 1A (TL1A)/TNF superfamily member 15 (TNFSF15) is a gut-associated proinflammatory cytokine originally characterized in a screen for TNF-α homologous molecules. It is expressed by activated T cells, dendritic cells, and monocytes and signals through death receptor-3 (DR3) (Meylan et al., 2008, Migone et al., 2002, Shih et al., 2009). TL1A is particularly relevant because it has previously been described as activating a subset of CD4^+^ memory T cells expressing IL-18Rα and DR3 (Holmkvist et al., 2015). More specifically, it has been shown to increase production of IFN-γ and TNF-α by CD161^+^CD4^+^ T cells in the presence of anti-CD3 or IL-12+IL-18 (Jin et al., 2013). This may be relevant to MAIT cell functions, because we have previously identified a phenotypic, functional, and transcriptional program shared by CD161-expressing cells (Fergusson et al., 2014).

Here, we addressed how TCR-dependent and TCR-independent signals synergize and drive the activation of *in vitro* blood- and gut-derived MAIT cells. We find that IL-12 and IL-18, in synergy with TCR triggering, promote the activation of MAIT cells and that additional TL1A signaling can optimize this response in a dose-dependent manner. Triggering with TCR alone or supported by cytokines drives a set of functions linked to a tissue-repair gene expression signature, accompanied by relevant protein expression and function. Overall, our data provide insight into the precise nature of TCR- and cytokine-mediated human MAIT cell activation, characterized by a range of effector functions including not only emergency host defense but also ongoing homeostasis maintenance. This feature may be relevant to other innate-like T cell subsets found at barrier sites in humans.

## Results

### TL1A and IL-15 Enhance Effector Functions of Human MAIT Cells

To explore the full impact of cytokine triggering of MAIT cells, we first examined extended combinatorial signaling. The ability of TL1A and IL-15 to promote T cell activation in the presence of a suboptimal IL-12 and IL-18 trigger has been shown in the CD161-expressing CD4^+^ T cells (Sattler et al., 2009, Cohavy et al., 2011, Jin et al., 2013, Holmkvist et al., 2015, Sattler et al., 2015). We therefore addressed whether MAIT cells possess similar responsiveness.

**Figure 1:**
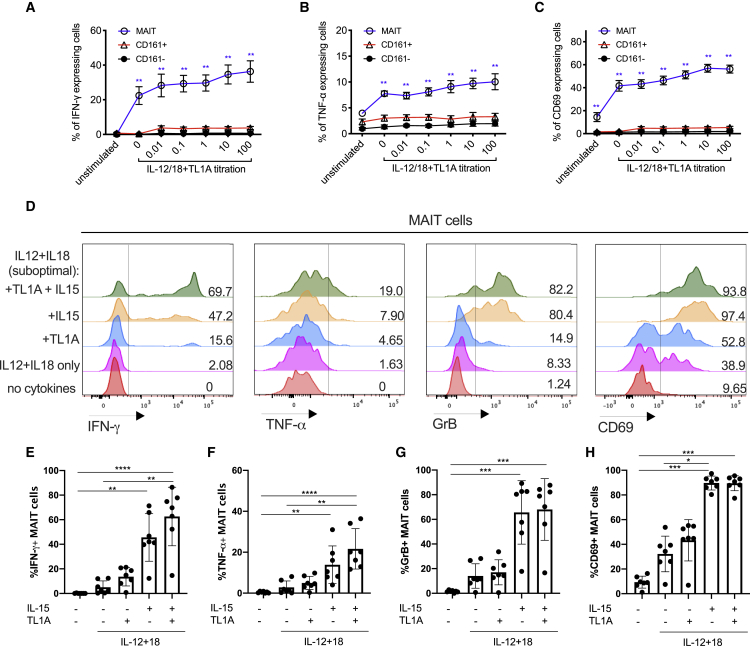
TL1A Enhances the Activation of MAIT Cells Suboptimally Stimulated with IL-12 and IL-18 CD8^+^ T cells were enriched from healthy peripheral blood mononuclear cells (PBMCs) and stimulated overnight with different combinations of cytokines: IL-12 at 2 ng/mL, IL-18 at 50 ng/mL, IL-15 at 25 ng/mL, and TL1A from 0.01 to 100 ng/mL as indicated. (A–C) Proportions of CD8^+^ MAIT/CD161^+^ or CD161^−^ cells producing IFN-γ (A), TNF-α (B), or CD69 (C) following overnight stimulation with suboptimal concentrations of IL-12 and IL-18, plus varying concentrations of TL1A. (D) Representative histograms showing the expression of IFN-γ, TNF-α, GrB, and CD69 by MAIT cells after stimulation with different combinations of cytokines. (E–H) Frequency of MAIT cells expressing IFN-γ (E), TNF-α (F), GrB (G), and CD69 (H) upon stimulation with the indicated cytokines. Data were acquired from seven donors in 2–3 experiments. Error bars represent means ± SEM. Differences among conditions were analyzed by Friedman tests with Dunn’s multiple comparison tests. ^∗^p < 0.05, ^∗∗^p < 0.01, ^∗∗∗^p < 0.001, ^∗∗∗∗^p < 0.0001. See also Figure S1.

TL1A triggered MAIT cell activation (as judged by expression of IFN-γ, TNF-α, and granzyme B) in combination with suboptimal doses of IL-12 and IL-18 in a dose-dependent manner (Figures 1A–1C). Expression of IFN-γ, TNF-α, and granzyme B by stimulated MAIT cells from a representative donor is shown in Figure 1D. IL-15 (50 ng/mL), TL1A (100 ng/mL), or the combination of both markedly promoted IL-12 (2 ng/mL)/IL-18 (50 ng/mL)-induced MAIT cell activation, measured by MAIT cell expression of IFN-γ, TNF-α, GrB, and CD69 (Figures 1E–1H).

Overall, we found that TL1A and IL-15 individually increased MAIT cell expression of IFN-γ and TNF-α and upregulated GrB and CD69 expression. IL-15 was more potent than TL1A when added singly to the IL-12+IL-18 culture, but the peak level of MAIT cell activation was achieved by a combination of both cytokines in the presence of IL-12 and IL-18. This combination was therefore used in downstream experiments. TL1A and IL-15 alone do not promote MAIT cell effector functions and have only a limited effect on CD161^+^ and CD161^−^ CD8^+^ T cells (Figure S1).

### MAIT Cells Respond to Combinations of Cytokines and TCR Triggering and Enhance Effector Functions in a Dose-Dependent Manner

**Figure 2:**
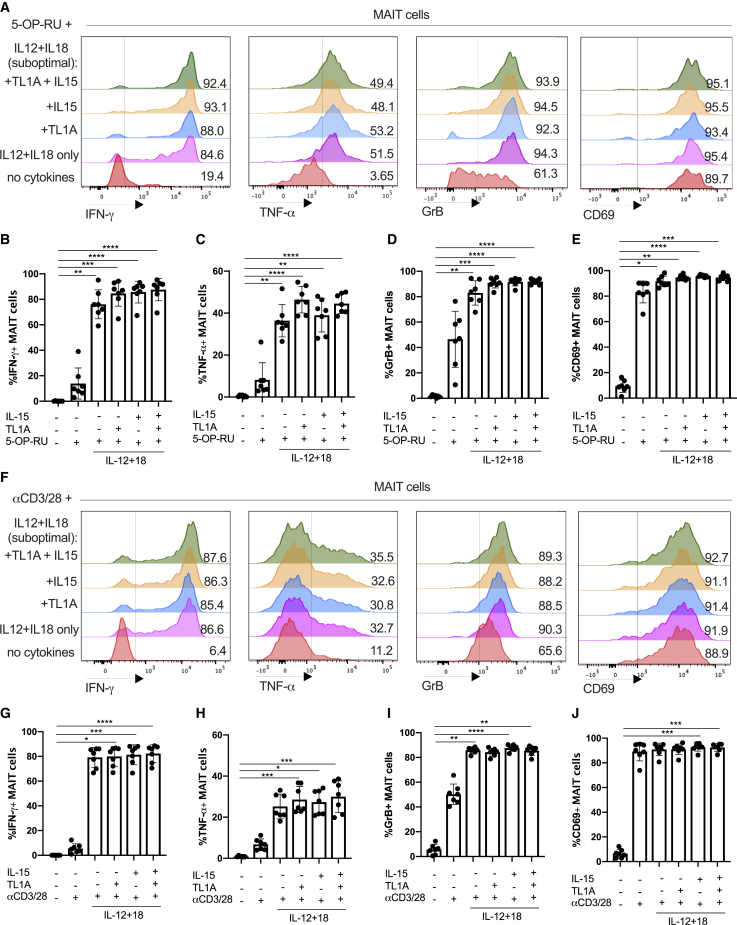
TCR and Cytokine Signaling Combine to Promote MAIT Cell Effector Functions (A–J) Magnetic-activated cell sorting (MACS)-enriched CD8 T cells from the blood were cultured overnight in the presence of the indicated cytokines, together with the THP1 cell pulsed with DMSO or the MAIT-antigen 5-OP-RU (A–E) or with αCD3/CD28 beads (F–J). (A) Representative histograms showing the expression of IFN-γ, TNF-α, GrB, and CD69 by MAIT cells after stimulation with different cytokines in the presence of 5-OP-RU. (B–E) Frequency of MAIT cells expressing IFN-γ (B), TNF-α (C), GrB (D), or CD69 (E) upon stimulation with the indicated cytokines. (F) Representative histograms showing the expression of IFN-γ, TNF-α, GrB, and CD69 by MAIT cells after stimulation with different cytokines in the presence of 5-OP-RU. (G–J) Frequency of MAIT cells expressing IFN-γ (G), TNF-α (H), GrB (I), or CD69 (J) upon stimulation with the indicated cytokines. Data were acquired from seven donors in two experiments. Error bars represent means ± SEM. Differences among conditions were analyzed by Friedman tests with Dunn’s multiple comparison tests. ^∗^p < 0.05, ^∗∗^p < 0.01, ^∗∗∗^p < 0.001, ^∗∗∗∗^p < 0.001. See also Figure S2.

Studies have addressed the hypo-responsiveness of CD8^+^ MAIT cells to anti-CD3 by comparing their ability to proliferate and produce cytokines *in vitro* to their CD161^−^ CD8^+^ counterparts (Turtle et al., 2011). We hypothesized that this could result from a lack of complementary inflammatory signals. Thus, we first asked how TCR and cytokine signaling combined (Figure 2). For these experiments, we used the optimized MR1 ligand 5-OP-RU and compared this to anti-CD3/CD28 bead stimulations.

Enriched CD8^+^ T cells were stimulated by suboptimal concentrations of IL-12 (2 ng/mL) and IL-18 (50 ng/mL) with or without TL1A and/or IL-15 in combination with 5-OP-RU (Figures 2A–2E). TCR signaling via 5-OP-RU had a profound synergy with added cytokines—with the major impact from IL-12 and IL-18—as measured by release of IFN-γ and TNF-α and upregulation of GrB, and CD69 (representative histograms in Figure 2A and combined data in Figures 2B–2E).

We repeated these protocols using anti-CD3/CD28 beads as the TCR trigger (Figures 2F–2J). The frequency of MAIT cells that responded to anti-CD3/CD28 by producing IFN-γ or TNF-α positively correlated with the bead-to-cell ratio (Figures S2A and S2B). Similar data were obtained to those using the 5-OP-RU trigger (histograms in Figure 2F and combined data in Figures 2G–2J). Again, in these combined TCR-cytokine stimulation studies, IFN-γ expression correlated with that of CD161, consistent with previous findings (Fergusson et al., 2015, Fergusson et al., 2014), and TL1A and IL-18 had no impact individually (Figure S2C).

**Figure 3:**
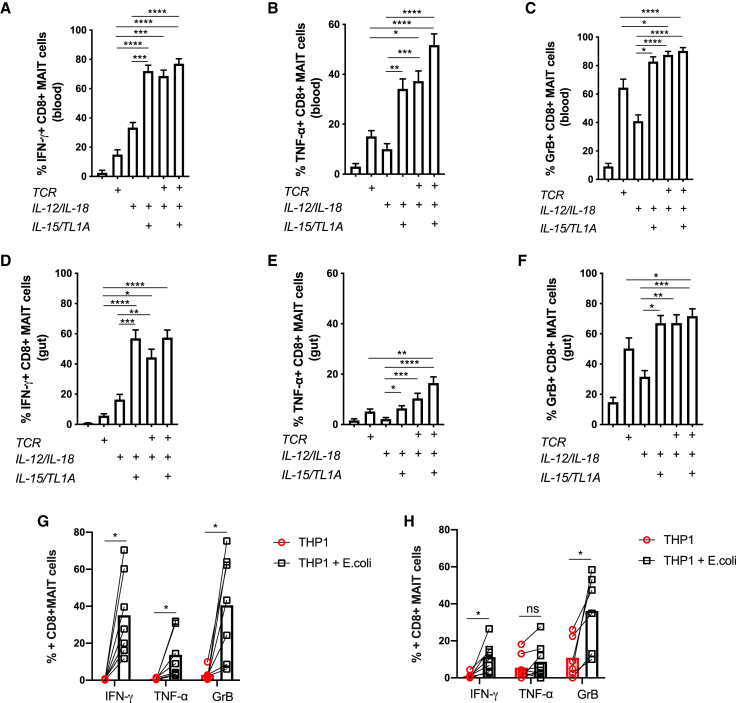
Gut-Derived MAIT Cells Show a Broadly Similar Response Pattern toward Innate and Adaptive Stimuli Compared with Their Blood-Derived Counterparts Representative plots showing the percentage of cells positive for the indicated effector molecules as a proportion of CD8^+^ MAIT cells. (A–C) Proportions of blood-derived (n = 32) CD8^+^ MAIT cells producing IFN-γ (A), TNF-α (B), or GrB (C) following overnight stimulation with combinations of suboptimal concentrations of IL-12 and IL-18, TL1A, and αCD3/CD28 beads as indicated. (D–F) Proportions of gut-derived (n = 13) CD8^+^ MAIT cells producing IFN-γ (D), TNF-α (E), or GrB (F) stimulated in the same way as in (A)–(C). (G and H) Expression of IFN-γ, TNF-α, and GrB by blood-derived (G, n = 7) or gut-derived (H, n = 6) CD8^+^ MAIT cells 20 h after coculture with THP1 cells alone or THP1 cells incubated with 25 fixed *E. coli* bacteria per cell. Data were acquired from multiple donors as indicated in 3–5 experiments. Error bars represent means ± SEM. Differences among conditions were analyzed by Friedman tests with Dunn’s multiple comparison tests (A–F), two-way ANOVA (G), or Wilcoxon tests (H). ^∗^p < 0.05, ^∗∗^p < 0.01, ^∗∗∗^p < 0.001, ^∗∗∗∗^p < 0.001. See also Figure S3.

In the periphery, MAIT cells can be exposed to various stimuli that could alter the way they respond to TCR and cytokine stimulation. To test to what extent the response patterns described earlier are preserved in barrier tissues, we analyzed MAIT cells isolated from the adjacent normal tissue taken at surgery for colonic cancer. The data may be affected by the presence of malignancy in the patients studied, and we have only examined broad patterns of responsiveness, rather than differences between tissues. In these tissue-derived cells, TCR and suboptimal IL-12/IL-18 triggers synergized strongly, and maximal activation was seen using combined stimulations, including IL-15 and TL1A, as was seen previously in blood-derived cells (Figure 3; Figure S3). We repeated these experiments with *E. coli* as a natural trigger of combined TCR- and cytokine-mediated stimulation (Figures 3G and 3H; Figure S3D), again observing a similar overall pattern of responsiveness.

### MAIT Cells Possess Distinct Transcriptional Signatures upon Activation by TCR or Cytokines

To explore the full breadth of effector functions of MAIT cells elicited by TCR or cytokine signals, we used RNA sequencing (RNA-seq) to characterize transcriptional profiles of MAIT cells under different treatments: TCR (anti-CD3/CD28, labeled here as T), cytokines (IL-12/IL-15/IL-18/TL1A, labeled here as C), and a combination thereof (labeled here as TC). Transcriptional profiles of differentially stimulated MAIT cells were compared with those of untreated (UT) cells. TCR beads were used at a 1:1 bead-to-cell ratio, and cytokines were used at the concentrations optimized earlier. To confirm activation, MAIT cells from the same donors were examined for their release of IFN-γ, TNF-α, and expression of GrB in response to the same stimulations (Figures S4A–S4C).

**Figure 4:**
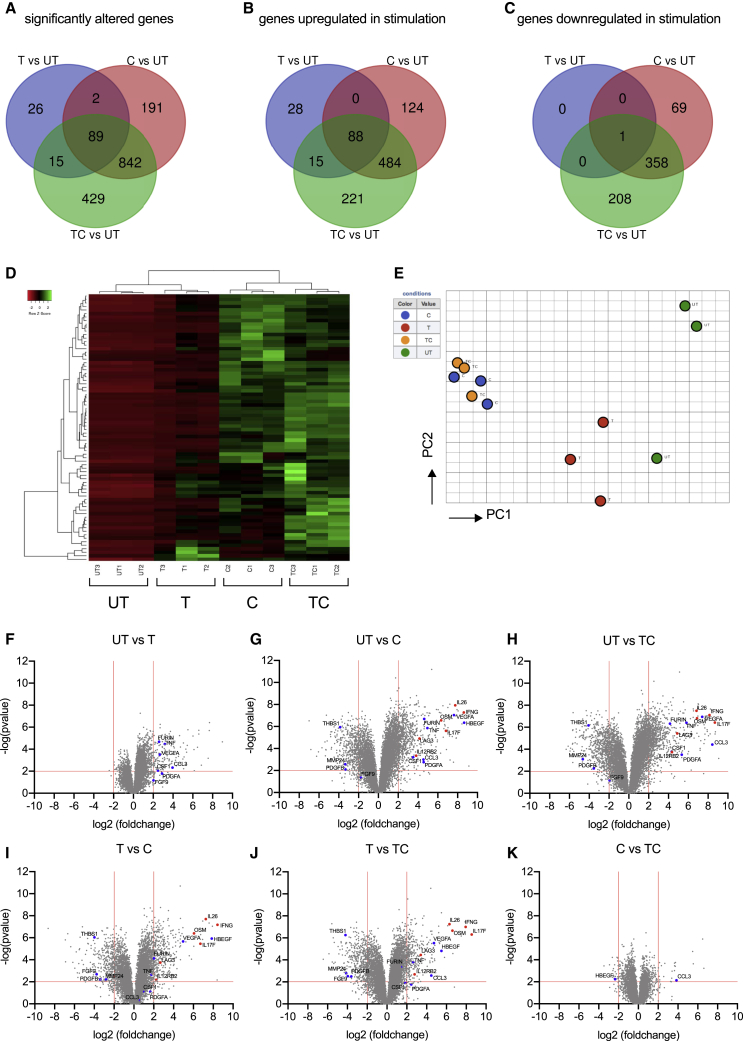
TCR- and Cytokine-Activated MAIT Cells Possess Distinct Transcriptional Profiles (A–C) Venn diagrams showing genes that are significantly differentially modulated (p < 0.05, fold change > 4) in TCR (T)-, cytokine (C)-, or TCR and cytokine (TC)-treated CD8^+^ MAIT cells compared with untreated (UT) MAIT cells of three healthy individuals. The cytokine (C) stimulation consisted of a cocktail of 4 cytokines: IL-12 (2 ng/mL), IL-18 (50 ng/mL), IL-15 (25 ng/mL), and TL1A (100 ng/mL). Genes with significantly altered expression levels (A) are divided into two sets: those are that are upregulated upon stimulation (B) and those that are downregulated upon stimulation (C). (D) Heatmap showing 1,594 significantly differentially expressed transcripts (p < 0.05, fold change > 4) between TCR/C/TC-stimulated and UT CD8^+^ MAIT cells among the same three healthy individuals. (E) Visualization of the CD8^+^ MAIT cell transcripts elicited by differential stimulations in the subspace of the first principle components (PCs). Each colored circle represents a sample and is color coded in accordance with the conditions with which cells were stimulated, as illustrated on the right-hand side of the graph. (F–K) Volcano plots to visualize differentially expressed transcriptional profiles of activated CD8^+^ MAIT cells stimulated in different ways. Each point represents a single gene, and genes expressed at significantly higher or lower levels between the compared conditions are depicted, respectively, in the upper-right or upper-left corner of each plot. Genes discussed in the text are highlighted in blue (tissue repair associated) or in red (inflammation associated). The gene expression of untreated MAIT cells was compared to (F) T-, (G) C-, or (H) TC-stimulated MAIT cells. Further, gene expression in those cells was also compared directly between the different stimulation conditions: (I) T- to C- stimulation, (J) T- to TC-stimulation, and finally (K) C- to TC-stimulation. Data were acquired from three donors in one experiment. See also Figure S4 and Tables S1, S2, and S3.

The mRNA levels of 132, 1,124, or 1,375 genes were significantly modulated (p < 0.01, |fold change| > 4, false discovery rate [FDR] < 0.05, including upregulation and downregulation) by TCR, cytokines, or combined TCR and cytokine stimulation, respectively. Venn diagrams highlight the overlapping and unique transcriptional signatures elicited by these 3 stimulations (Figures 4A–4C; Table S1). We found that stimulating MAIT cells with TCR beads and/or cytokines resulted in significant alteration of 89 common mRNA transcripts in MAIT cells, consisting of 88 upregulated genes (Figure 4B; Table S1) and 1 downregulated gene (Figure 4C). Gene Ontology (GO) enrichment analysis on these common 88 upregulated genes by MAIT cells predicted that they are involved in C production and signaling, including, of relevance, IL-12-mediated signaling (IL23R, EBI3, IL2RA, RELB, NFKB1, NFKB2, and CCL3), and TNF signaling (TNF, NFKB1, and NFKBIA).

Analysis of the other genes unique to TCR and cytokines (TC) indicates these two signals induce diverse physiological functions in MAIT cells (Figure 4A). Among 1,594 modulated genes (Table S2), 960 (60.2%) were upregulated and the rest were downregulated (Figures 4A and 4B). MAIT cells stimulated via TC shared 572 upregulated genes with their counterparts that were only stimulated with cytokines. They constitute 82.1% of upregulated gene transcripts elicited by cytokines and 70.8% elicited by TCR beads and cytokines (Figure 4B).

The 1,594 genes with significantly altered expression levels among conditions were then plotted in a heatmap according to their normalized expressions using average linkage hierarchical clustering (Figure 4D). We also performed a principal-component analysis (PCA) using the first two principal components of the 1,594 mRNA transcripts and visualized the correlation of the transcriptional profiles of differentially stimulated MAIT cells (Figure 4E). Clustering of C− and TC− conditions confirmed that cytokine stimulation at this time point had a dominant impact on MAIT cell activation. However, the finding of 221 upregulated genes (Figure 4B) and 208 downregulated genes (Figure 4C) unique to TC stimulation also suggests a strong synergy between TCR signaling and cytokines to drive MAIT cell activation and promote their effector functions. Furthermore, the PCA analysis indicates that a TCR stimulus alone can trigger a pronounced level of activation, because TCR clustering was clearly separate from UT.

We next analyzed volcano plots that show differentially expressed transcriptional profiles of stimulated MAIT cells compared with their unstimulated counterparts (Figures 4F–4H) and compared among stimulations (Figures 4I–4K). Overall, a more limited number of genes were significantly altered by a single dose of TCR stimulation, after filtering for transcripts with p < 0.01 and fold change > 4 (Figure 4F), compared with more dynamic transcriptional profiles seen following stimulation with cytokines (Figures 4G and 4H). The transcriptional impact of cytokines on top of TCR stimulation (TCR versus TC) is shown in Figure 4J.

To confirm these findings from RNA-seq, we first used qPCR to validate 3 of the most highly upregulated genes—*IL-26*, oncostatin M (*OSM*), and heparin binding early growth factor (*HBEGF*) (Figures 4G and 4H)—on RNA samples extracted from activated MAIT cells (Figures S4E–S4G). In all 3 cases, we demonstrate a similar pattern of responsiveness by an independent method. Overall, these data indicated that a range of responses can be generated by MAIT cells in response to TCR and cytokine triggers and that the pattern of these differ between the triggers used. We therefore went on to explore the significance of this in more depth.

### Transcriptional Signatures of Activated MAIT Cells Predict Not Only Antimicrobial but also Tissue-Repair Functions

Given the range of responses seen after TCR- and cytokine-mediated activation, we speculated that functions of MAIT cells extended beyond conventional antimicrobial responses. The discovery of a skin-homing Tc17 subset in mouse responsive to commensal ligands has shed light on a unique form of adaptive immunity in which antimicrobial functions and tissue repair are coupled within the same subset of unconventional T cells (Harrison et al., 2019, Linehan et al., 2018). These commensal-specific T cells elicited a tissue-repair signature and accelerated wound closure, in addition to promoting protection against pathogens. MAIT cells are commensal responsive and similarly have been associated with a type-17 phenotype (Billerbeck et al., 2010, Dusseaux et al., 2011, Sobkowiak et al., 2019). Therefore, we investigated functional overlap using a genomic comparison between activated human MAIT cells and mouse skin-homing Tc17 cells.

First, we examined the volcano plots in Figures 4F–4K and annotated the genes from the tissue-repair gene list used in the study of Linehan et al. (2018) (Table S3). The genes on these plots (Figures 4F–4K) are color coded according to whether they associated with a proinflammatory and an antimicrobial response, as has been classically associated with MAIT cells (red) or tissue-repair signature (blue). Substantial numbers of genes linked with the tissue-repair signature were observed, including genes such as Furin, TNF, CSF1, and CCL3 and various growth factors.

**Figure 5:**
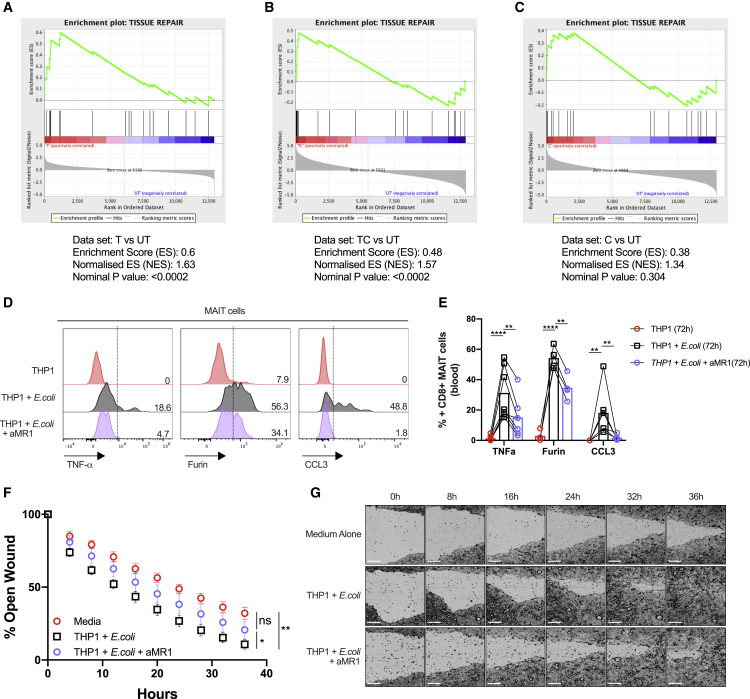
TCR-Mediated Activation of MAIT Cells Leads to the Expression of Tissue-Repair-Associated Molecules and Accelerates Wound Healing (A–C) Gene set enrichment summary plots for stimulated sorted MAIT cell-versus-unstimulated cell-ranked genes. Depicted are the individual plots for TCR-stimulated versus UT in (A), TC-stimulated versus UT in (B), and C versus unstimulated in (C). Non-significant for C versus UT, normalized enrichment score (NES) = 1.63; p < 0.0002 for TCR versus UT, NES = 1.57; and p < 0.0002 for TC versus UT. Data were acquired from three donors in one experiment. (D) Flow cytometry analysis of the expression of TNF-α, furin, and CCL3 by CD161^++^/MAIT CD8^+^ T cells in response to fixed *E. coli* presented by THP1 cells in the presence or absence of an anti-MR1 (αMR1) blocking antibody at the 72-h time point. (E) Statistical analysis of the expression of the effector molecules shown in (D). (F) Caco2 cells were grown to confluency and scratched with a WoundMaker device to perform *in vitro* wound-healing assays. Cells were supplemented with different supernatants collected from 72-h cocultures of enriched CD8 T cells with *E. coli*-loaded THP1 cells in the presence or absence of αMR1, as indicated. The open wound areas were quantified as percentages of the initial wound size in the Caco2 cultures. Data points are mean ± SEM and were acquired from five biological replicates in two experiments. (G) Representative pictures of the closure of the wounds in Caco2 cultures treated as in (F) were assessed with time-lapse imaging over a time course of 36 h. Data were acquired from seven donors in three experiments. Differences among conditions were analyzed by two-way ANOVA. ns, not significant; ^∗^p < 0.05, ^∗∗^p < 0.01, ^∗∗∗∗^p < 0.001. Scale bars, 250 μm. See also Figure S5 and Table S3.

Next, genes that were significantly differentially expressed compared with unstimulated MAIT cells were identified from TCR-, C-, and TC-stimulated MAIT cells and statistically compared in aggregate to the tissue-repair gene dataset (Linehan et al., 2018). Gene set enrichment analysis demonstrated significant enrichment (p < 0.0002) of these tissue repair-related genes in MAIT cells stimulated by TCR with or without cytokines (Figures 5A and 5B), but not by cytokines alone (Figure 5C). The significant leading edge genes from these analyses are indicated in Figures S5A and S5B. These data suggest that TCR triggering by MAIT cells may be important in driving a tissue-repair program.

### Examination of MAIT Cell Functions Confirms a Tissue-Repair Activity

To test these findings, we analyzed the expression of 3 of these genes from the tissue-repair signature on the protein level by flow cytometry. Triggering of MAIT cells by *E. coli* led to the production of TNF, Furin, and CCL3 in a TCR-dependent manner, because it could be blocked fully or partially by anti-MR1 at 20 or 72 h, respectively (Figures 5D and 5E; Figures S5C and S5D). We also validated upregulation of granulocyte-monocyte colony-stimulating factor (GM-CSF; CSF2), a tissue-repair-associated gene not upregulated at earlier time points, which again was most evident in extended cultures (72 h) and triggered in an MR1-dependent fashion (Figures S5E and S5F).

To assess this, we used *in vitro* wound-healing assays, combining the intestinal epithelial cell line Caco2 (see STAR Methods) (Povoleri et al., 2018) with supernatants derived from MAIT-containing CD8^+^ T cell cultures stimulated with *E. coli* in the presence or absence of anti-MR1 blocking antibodies for 72 h. Supernatants obtained from *E. coli*-stimulated CD8^+^ T cell cultures significantly accelerated wound closure in this system, which was most evident at later time points (e.g., 24–36 h) (Figure 5G). This effect was significantly reduced when MR1 was blocked, underscoring the importance of MR1-dependent TCR signaling in the process (Figures 5F and 5G). Altogether, these data provide further evidence that TCR-dependent activation is essential for the expression of tissue-repair-associated molecules by MAIT cells and that it, in principle, allows MAIT cells to affect key aspects of tissue repair like the migration and/or proliferation of epithelial-type cells.

### Comparative Analyses of Human and Mouse MAIT and Tissue-Repair Datasets

**Figure 6:**
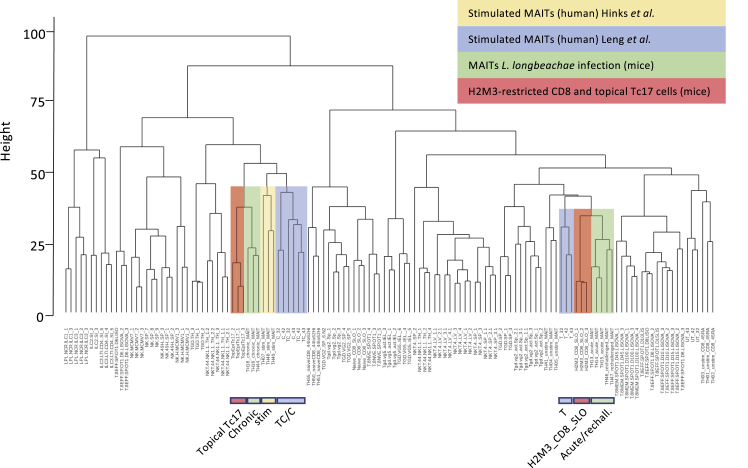
Integrated Transcriptional Analyses Reveal the Relationship between *In Vitro*-Activated Human MAIT Cells and *In Vivo*-Activated Murine MAIT and Tc17 Cells Hierarchical clustering analysis of the transcriptomic profiles of the indicated cell populations is shown. Similarity between the expression profiles is measured using a Euclidean distance (height). Datasets were derived from ImmGen (Heng et al., 2008), Linehan et al. (2018), and Hinks et al. (2019) and were integrated as described in the STAR Methods section. The relevant datasets are colored. UT, TCR, C, and TC refer to the conditions used in this paper on *in vitro*-activated human CD8^+^ MAIT cells (blue). Topical Tc17 and H2M3_CD8_SLO refer to the H2M3-restricted populations identified in Linehan et al. (2018) in the skin and secondary lymphoid organs of mice, respectively (red). The cells described in Hinks et al. (2019) are marked in yellow (stimulated human MAIT cells) or green (chronic or acute, derived during a late or an early time point after *L. longbeachae* infection in mice, respectively). See also Figure S6.

We performed a data integration analysis by fusing RNA-seq datasets containing mouse data from activation studies *in vivo* and *in vitro* (Hinks et al., 2019) with our human data and applying a protocol for such integration (Marchi et al., 2019). First, we examined how our data aligned with those obtained by Hinks et al. (2019), who examined mouse MAIT cell activation *in vitro* (5-OP-RU stimulation) and *in vivo* (bacterial challenge), because these were shown to align with activated H2M3-restricted cells from publicly available data from Linehan et al. (2018) (Figure 6). The accompanying dendrogram shows the close transcriptional relationship between our *in vitro*-activated human T cells and those in the mouse. In this analysis, the closest neighbors of the maximally activated cells (TC and C) were the Tc17 cells activated in the mouse skin and the *in vivo* chronically or *in vitro*-activated MAIT cells from Hinks et al. (2019). In the less activated condition (TCR), the human MAIT cells from our study cluster with H2M3-restricted CD8 T cells from the murine secondary lymphoid organs, which were shown to be less capable of cytokine production compared with their skin-derived counterparts and with murine MAIT cells acutely activated *in vivo*. Hence, despite the cross-species comparison, stronger stimulation creates an important and relevant shift in the position on the dendrogram. These data indicate, in an unsupervised analysis, that closely shared transcriptional patterns exist between our *in vitro*-stimulated cells and different subsets that are performing tissue-repair functions (as well as host defense functions) *in vivo* in mice.

Finally, we performed, using an extension of this bioinformatic approach, alignment among all 3 generated datasets on human and mouse MAIT cell activation to assess their comparability. All 3 have shown that under conditions in which the TCR is stimulated, there is a tissue-repair signature, but when tested in both our dataset and that of Lamichhane et al. (2019), the statistical association in gene set enrichment analysis (GSEA) studies was lost under conditions of cytokine stimulation alone. In Figure S6, all unstimulated human MAIT cells clustered (indicating both good data fusion and consistency between studies), with a large block of long-term activated mouse and strongly activated human MAIT cells sitting in a distinct clade. In this analysis, we could split TCR and UT conditions, and the TCR conditions clustered closely with the acutely activated murine MAIT cells, indicating even this relatively modest response in our assay conditions parallels a transcriptional state observed *in vivo*. Overlap was seen between the different human datasets (e.g., between bacterial- and ligand-stimulated cells from different sources), indicating the comparability of the studies despite the different protocols and sites.

### A Local Model for MAIT Cells in Epithelial Defense and Tissue Repair

**Figure 7:**
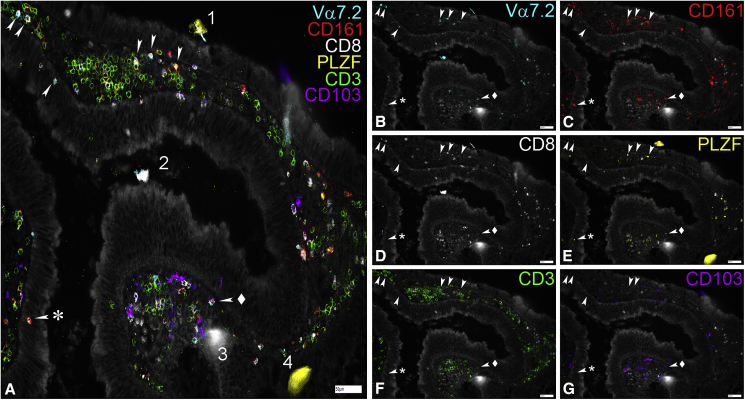
MAIT Cells Can Be Found Close to and within the Colonic Epithelium (A–G) Representative images showing the expression of Va7.2, CD161, CD8, PLZF, CD3, and CD103 in the lamina propria and the epithelium of fixed samples of colonic polyp tissue. Samples were mounted on cytometer chips and iteratively stained with sets of three directly fluorochrome-conjugated antibodies as described in the methods section. Depicted are a merged picture (A) and all the individual stains for Va7.2 (B), CD161(C), CD8 (D), PLZF (E), CD3 (F), and CD103 (G). White arrows mark cells showing co-expression of Va7.2, CD161, PLZF, and CD3 that were defined as MAIT cells here. Note that while CD8 was co-expressed in most of them, CD8− MAITs (arrow + asterisk) could also be found. In contrast, CD103 was rarely co-expressed on MAITs (arrow + diamond). During the iterative staining process dust particles and other detritus can be picked up by the solution flowing over the tissue creating autofluorescent artifacts (1–4). While some of these get washed away after completion of the staining cycle (1, 4), others present during multiple imaging rounds (2, 3). Scale bars, 50μm.

Given the relationship between MAIT cells and epithelial maintenance, we wished to assess how closely associated such cells are with the epithelial layer. Because only limited data are available on this (partly because of the availability of suitable antibodies and the need for antibody combinations to reliably identify MAIT cells), we developed a high content imaging protocol based on chip cytometry. By costaining for multiple relevant markers (CD3, CD8, CD161, Vα7.2, and PLZF) in colonic tissue, we could observe apposition between MAIT cells and intact epithelium, suggesting two-way cross talk is possible under homeostatic conditions (Figure 7).

## Discussion

CD161-expressing human TCR lymphocytes possess shared transcriptional and functional phenotypes, and their enhanced innate ability to respond to inflammatory cues has been investigated with transcriptomic approaches (Fergusson et al., 2014). MAIT cells comprise a large proportion of these CD161-expressing T cells and have previously been described as showing limited responses to conventional TCR signals, although combinatorial signaling can markedly augment this *in vitro* and *in vivo* (Slichter et al., 2016, Turtle et al., 2011) (see also accompanying papers by Hinks et al., 2019, and Lamichhane et al., 2019). However, MAIT cells can respond in a fully TCR-independent manner, via cytokine signaling, and such behavior can trigger protection *in vivo* (Wilgenburg et al., 2018). The functional consequences of TCR-dependent versus TCR-independent activation of MAIT cells have not been fully defined; thus, dissecting the differential signals that promote and sustain MAIT cell effector function is of central importance in defining the role of MAIT cells in both health and disease. Here we probed the contribution of innate and adaptive signals to MAIT cell activation in the blood and gut, describing segregating functions of MAIT cells in response to different activation stimuli.

We defined MAIT cells in both human blood and human gut as CD161^2+^Vα7.2^+^CD8^+^CD4^−^CD3^+^ T cells, a common approach used by several studies to dissect MAIT cell effector function elicited by cytokines or the TCR (Kurioka et al., 2015, Kurioka et al., 2017, Sattler et al., 2015, Ussher et al., 2014b, van Wilgenburg et al., 2016). MR1 tetramers, combined with CD161 staining, identify MR1-restricted MAIT cells unequivocally, but these reagents only became available at the end of this study. We also stained freshly isolated colonic lymphocytes using the MR1 tetramer, with Vα7.2 and CD161 antibodies on the same panel. These provided similar estimates of frequency (as they commonly do in blood). An example is shown in Figure S3C.

Responses following a TCR stimulation of MAIT cells and conventional CD8 T cells differ in magnitude and in quality. We and others (Slichter et al., 2016) initially explored this using bead-based protocols. This allowed a direct comparison with non-MAIT populations and simplified some downstream sorting procedures. Although CD3/CD28 beads do not represent a true physiological TCR stimulus, here we confirmed the bead-based data using 5-OP-RU, an optimized MR1 ligand, and *E. coli* stimulation, which represents a more physiological trigger. In each case, there is clearly activation in response to TCR triggering, but this is markedly amplified and sustained through combinatorial signals via cytokines. These broad features were recapitulated in MAIT cells derived from the human gut.

Resting MAIT cells express a higher level of IL-18Rα on the cell surface compared with their CD161^2+^Vα7.2^−^ or CD161^−^ counterparts (Fergusson et al., 2014, Ussher et al., 2014b). However, we and others found that IL-18 alone has a limited role in activating lymphocytes, including MAIT, natural killer (NK), and T cells (Tominaga et al., 2000, Sareneva et al., 2000). A signal combining IL-12 and IL-18 has been described as activating a range of lymphocytes, including T helper 1 (Th1) cells, B cells, NK cells, and more recently, MAIT cells, independently from the TCR. Expanding on this, our data also show TL1A can synergize with these cytokines to enhance MAIT cell activation. In addition to its proinflammatory and costimulatory role in the human blood, TL1A is a gut-associated cytokine and has been linked to inflammatory bowel disease (IBD) (Jin et al., 2013, Shih et al., 2009). Patients with IBD have higher levels of DR3 and TL1A expression in their mucosal T cells and macrophages (Bamias et al., 2003, Prehn et al., 2004). We highlight that TL1A enhances the effector function of gut MAIT cells in the presence of a suboptimal dose of IL-12+IL-18, suggesting that TL1A may contribute to the amplification of inflammatory responses. Thus, TL1A blockade *in vivo* could potentially achieve an anti-inflammatory response while maintaining barrier function. These data confirm and extend the importance of TNF superfamily members in MAIT cell activation—as revealed for TNF-α in responses to opsonized bacteria at limiting doses (Banki et al., 2019)—and suggest a context-specific and antigen-presenting cell (APC)-dependent role for these signals.

Genes controlling the TCR signaling pathway have been shown to be differentially regulated in MAIT cells compared with conventional CD8^+^ T cells (CD161^−^ cells) (Turtle et al., 2011), yet full implications of a partial response by TCR-triggered MAIT cells have been unclear so far. We therefore explored this using RNA-seq of MAIT cells stimulated *in vitro* via TCR-dependent and TCR-independent pathways. Our RNA-seq data reveal both shared and independent response patterns between TCR-dependent and TCR-independent stimulation. Although the magnitude of change seen in our experiments was greater in the cytokine-stimulated cells, the transcripts associated with TCR triggering alone provided a clear insight into potential function. We linked, using GSEA, the MAIT TCR-driven transcriptional profile with a tissue-repair signature from a report on IL-17+ innate-like CD8^+^ T cells in a murine skin model (Linehan et al., 2018) (later confirmed in the gut by Harrison et al., 2019). The tissue-repair profile in the unconventional (H2M3-restricted) mouse skin CD8^+^ T cells was shown to be linked to an encounter with commensal microbes and to affect cutaneous wound healing. In our model, we have shown a functional impact of TCR-dependent microbe-triggered MAIT cells *in vitro* using a monolayer scratch assay.

We validated expression of some of the most critical genes underpinning this signature by MAIT cells on the protein level. These genes included furin, which plays an important role in tissue repair through its broad proprotein convertase activity, leading to activation of proteins like transforming growth factor β (TGF-β) and matrix metalloproteinases. T cell-derived furin has been shown to be critical in tissue protection in a transfer colitis model and affects regulatory T cell (Treg) development (Pesu et al., 2008). CCL3 (MIP1α), which is more broadly expressed and has been better studied in T cells, has a critical role in macrophage recruitment (DiPietro et al., 1998). Both of these mediators and TNF-α, which has a described role in tissue repair in concert with CCL3 (Li et al., 2016), were produced by activated MAIT cells in a sustained and TCR-dependent manner. The repair signature was most evident in MAIT cells triggered via their TCR, either with or without cytokines, but there was no statistical enrichment in the cytokine-only stimulation. This is most evident in Figures 4F–4K, in which some key genes are marked. For example, the cytokine-alone stimulus induces many relevant genes, but there is a slight abundance of inflammatory and host defense genes upregulated in the cytokine versus TCR comparison (Figures 4I versus 4F), together with some downregulation of tissue-repair genes from the GSEA list (Table S3). These findings were corroborated by the results of an *in vitro* wound-healing assay, because blocking MR1-dependent TCR signaling in CD8 T cell cultures abolished the accelerating effect that these supernatants otherwise had on wound closure (Figures 5F and 5G).

Beyond the list of tissue-repair factors identified in the work of Linehan et al. (2018) and used in the GSEA comparison, other MAIT cell-derived factors were found through the RNA-seq study (and validated in independent assays), with potential roles in inflammation and in tissue homeostasis. For example, IL-26 is part of the IL-20 family of cytokines (including IL-22, also made by MAIT cells; Gibbs et al., 2017), which all strongly affect epithelial cell function, including wound repair (Rutz et al., 2014). Similarly, OSM, which is upregulated in the gut during inflammation *in vivo* (West et al., 2017), has been shown to induce migration of keratinocytes *in vitro* and skin repair *in vivo* (Boniface et al., 2007, Hoffmann et al., 2011), while HBEGF, which was highly expressed by activated MAIT cells, has a long track record in tissue regeneration (reviewed in Dao et al., 2018). Altogether, these data substantially broaden the known functions of MAIT cells and include a range of functions on a spectrum of host defense, inflammation, and barrier repair.

A similar set of RNA-seq data, functional data, and conclusions has been obtained using parallel experiments in human MAIT cells *ex vivo* with a 5-OP-RU and bacterial trigger and most importantly in an *in vivo* challenge incorporating a TCR trigger with and without cytokines (Hinks et al., 2019, Lamichhane et al., 2019). Several observations can be made from a comparison of the transcriptional data. First, in all 3 cases, a GSEA analysis using TCR-triggered MAIT cells revealed a statistically robust congruence with the tissue-repair signature. In both studies in which this was addressed, this was not seen with cytokine stimulation alone (IL-12/IL-18 in the parallel study) (Lamichhane et al., 2019). Looking more broadly at transcriptional regulation of MAIT cells in mouse and man, the data fusion analyses also showed a strong link between the activation of human MAIT cells shown here (and in parallel studies) and the Tc17 tissue-repair subset of Linehan et al. (2018). This is an interesting, and we feel important, extension of the work, allowing a direct comparison between *in vivo* mouse data and multiple parallel sets of *in vitro*-stimulated human data across species, platforms, experimental protocols, and sites. Because this is an unsupervised and hypothesis-free approach, it lends weight to the associations seen here and the triggering model we have produced. We propose that it provides a useful template for future studies of such populations for which the aggregation of data enhances its biological impact (as well as providing independent validation). Thus, a reasonable body of data has emerged in parallel among the three studies that suggests MAIT cells possess tissue-repair activity of relevance in barrier defense.

Taking together the data here and those of Hinks et al. (2019) and Lamichhane et al. (2019), we propose a model whereby in the human gut and potentially in the liver, MAIT cells are continuously exposed to MR1-bound ligand derived from the commensal bacteria present in the microbiome. In the absence of inflammatory signals, this only drives the circumscribed transcriptional signature associated with local homeostatic function. This model would fit well with emerging data that in tissues such as the gut, MAIT cells can play a protective role. This is most clearly seen in the study of non-obese diabetic (NOD) mice, in which the genetic absence of MR1 leads to loss of mucosal integrity and bacterial translocation in the steady state (Rouxel et al., 2017). A similar epithelial protective role is seen in a model of graft versus host disease (Varelias et al., 2018). This adds to existing data indicating a classical immune protective role against infection, for example, in the lung, following bacterial challenge (Wang et al., 2018). During loss of bowel integrity associated, for example, with IBD, MAIT cells have been shown to be further activated in some studies (Serriari et al., 2014), but whether this is a response to tissue injury or they play a causative role has yet to be defined. Our data suggest that during tissue injury, the innate cytokines induced would drive the full activation signal seen, which includes a broad inflammatory response. For example, IL-17F, which has been shown to be pathogenic in IBD models (Tang et al., 2018), is not induced by TCR signals alone but is strongly induced in the presence of inflammatory cytokines (fold change > 400, p < 10^−6^).

There are some limitations to the study presented. This includes the analysis of gut tissue, in which the material was obtained from patients with a colonic tumor, potentially affecting uninvolved mucosa. The study also focused on the majority CD8^+^ MAIT cells. Their triggering behavior reflects that of other MAIT subsets in which this has been studied, although some functional differences may exist (Kurioka et al., 2017). Analysis of transcriptional changes was limited to a single time point, and analysis of shorter and longer stimulations could prove impactful. In such studies, it would also be of interest to specifically address the influence of both age and gender, which were not studied in this analysis because of the anonymized nature of the blood cones. Finally, the analysis of MAIT cell location using high content cytometry (Figure 7) was limited to intact epithelium and should be extended in future studies to identify the transcription factors and cytokines expressed both in the steady state and in an inflammatory state to test these ideas *in situ*.

Overall, we have defined combinatorial pathways to activate MAIT cells, extending the role of TNF superfamily members such as TL1A, and we have dissected the consequences of activation via TCR-dependent and TCR-independent pathways in human blood and gut. Given the overlap between tissue repair and host defense and inflammatory programs found in MAIT cells (and related populations in the mouse), our model suggests that ongoing maintenance of the barrier is an integral part of the function of such unconventional cells concentrated in epithelia, which goes hand-in-hand with control of microbial invasion.

## STAR★Methods

### Key Resources Table

**Table:** 

REAGENT or RESOURCE	SOURCE	IDENTIFIER
**Antibodies**		

LEAF-purified mouse anti-human CD3 (clone UCHT1)	Biolegend	Discontinued Alternative: Cat# 300438; RRID: AB_11146991
LEAF-purified mouse anti-human CD28 (clone CD28.2)	Biolegend	Cat# 302914; RRID: AB_314316
LEAF-purified mouse anti mouse/rat/human MR1 (clone 26.5)	Biolegend	Cat# 361103; RRID: AB_2563041
Anti-human CD3 (clone UCHT1) PerCp/Cy.5	Biolegend	Cat# 300428; RRID: AB_893298
Anti-human CD3 (clone OKT3) eFluor450	eBioScience	Cat# 48-0037-42; RRID: AB_1272055
Anti-human CD3 (clone OKT3) BV605	Biolegend	Cat# 317321; RRID: AB_11126166
Anti-human CD4 (clone M-T466) VioGreen	Miltenyi	Cat# 130-113-259; RRID: AB_2726060
Anti-human CD4 (clone OKT4) PerCP/Cy5.5	eBioScience	Cat# 45-0048-42; RRID: AB_10804390
Anti-human CD8 (clone SK1) FITC	Biolegend	Cat# 344704; RRID: AB_1877178
Anti-human CD8 (clone REA734) VioGreen	Miltenyi	Cat# 130-110-684; RRID: AB_2659245
Anti-human CD8 (clone REA734) PE-Vio770	Miltenyi	Cat# 130-110-680; RRID: AB_2659245
Anti-human CD39 (clone A1) PE	Biolegend	Cat# 328207; RRID: AB_940427
Anti-human CD69 (clone H1.2F3) eFluor450	eBioScience	Cat# 48-0691-82; RRID: AB_10719430
Anti-human CD103 (Ber-ACT8) PE	Biolegend	Cat# 350206; RRID: AB_10641843
Anti-human CD161 (clone 191B8) APC	Miltenyi	Cat# 130-113-590; RRID: AB_2733346
Anti-human CD161 (clone 191B8) PE	Miltenyi	Cat# 130-113-593; RRID: AB_2733772
Anti-human CD161 (clone 191B8) PE-Vio770	Miltenyi	Cat# 130-113-594; RRID: AB_2751134
Anti-human CCL3/(4) (clone 93342) APC	R&D Systems	Cat# AF270NA; RRID: AB_354436
Anti-human Furin (clone 222722) AF647	R&D Systems	Cat# IC1503R-100UG; https://www.rndsystems.com/products/human-furin-alexa-fluor-647-conjugated-antibody-222722_ic1503r
Anti-human GM-CSF (clone BVD2-21C11) PerCP/Cy5.5	Biolegend	Cat# 502312; RRID: AB_11147946
Anti-human GrB (clone GB12) APC	Initrogen	Cat# MHGB05; RRID: AB_1500190
Anti-human GrB (clone GB11) AF700	BD BioSciences	Cat# 561016; RRID: AB_2033973
Anti-human IFNγ (clone 4S.B3) AF700	Biolegend	Cat# 502520; RRID: AB_528921
Anti-human IFNγ (clone 45-15) FITC	Miltenyi	Cat# 130-091-641; RRID: AB_244194
Anti-human IFNγ (clone 4S.B3) PE/Cy7	Biolegend	Cat# 502528; RRID: AB_2123323
Anti-human IgG2bκ (clone 133303) AF647	R&D Systems	Cat# IC0041R; RRID: AB_2737095
Anti-human PD-1 (clone EH12.2H7) BV421	Biolegend	Cat# 329920; RRID: AB_10960742
Anti-human/mouse PLZF (clone R17-809) PE	BD Pharmigen	Cat# 564850; RRID: AB_2738984
Anti-human TNFα (clone Mab11) FITC	Biolegend	Cat# 502906; RRID: AB_315258
Anti-human TNFα (clone Mab11) PerCP/Cy5.5	Biolegend	Cat# 502926; RRID: AB_2204081
Anti-human Vα7.2 (clone 3C10) APC	Biolegend	Cat# 351708; RRID: AB_10933246
Anti-human Vα7.2 (clone 3C10) FITC	Biolegend	Cat# 351704; RRID: AB_10900975
Anti-human Vα7.2 (clone 3C10) PE	Biolegend	Cat# 351710; RRID: AB_2561954
Anti-human Vα7.2 (clone 3C10) PE/Cy7	Biolegend	Cat# 31712; RRID: AB_2561994
Anti-human TCRγ/δ (clone IMMU510) FITC	Beckman Coulter	Cat# IM1571U; https://www.mybeckman.uk/reagents/coulter-flow-cytometry/antibodies-and-kits/single-color-antibodies/tcr-pan-g-d/im1571u
Anti-human TCRγ/δ (clone 11F2) APC-Vio770	Miltenyi	Cat# 130-113-501; RRID: AB_2751120

**Bacterial and Virus Strains**		

*E.coli* DH5α	Invitrogen	Cat# 18265017

**Biological Samples**		

Leukocyte cones	NHS Blood and Transplant	https://www.nhsbt.nhs.uk/
Patient-derived resections (CRC)	TGU Biobank	https://www.expmedndm.ox.ac.uk/tgu/tgu-biobank-ibd-cohort
Patient-derived resection (IBD)	TGU Biobank	https://www.expmedndm.ox.ac.uk/tgu/tgu-biobank-ibd-cohort

**Chemicals, Peptides, and Recombinant Proteins**		

Brefeldin A solution (1000X)	eBioScience	Cat# 00-4506-51
5-OP-RU	Fairlie group, Mak et al., 2017	N/A
Collagenase A	Roche (mft)/ Merck	Cat# 10103578001
DNase I	Roche (mft)/ Merck	Cat# 11284932001
SuperScript III Reverse Transcriptase	Invitrogen	Cat# 18080093
Human IL-12, premium grade	Miltenyi	Cat# 130-096-705
Human IL-15, premium grade	Miltenyi	Cat# 130-095-764
Recombinant Human IL-18	MBL	Cat# B001-5
Recombinant Human TL1A/TNFSF15	R&D Systems	Cat# 1319-TL-010

**Critical Commercial Assays**		

T cell Activation/Expansion Kit, human	Miltenyi	Cat# 130-091-441
RNeasy Micro Kit	Quiagen	Cat# 74004
LIVE/DEAD Fixable Near IR Dead Cell Stain Kit	Invitrogen	Cat# L10119
Permeabilization buffer (10x)	eBioScience	Cat# 00-8333-56
CD8 MicroBeads, human	Miltenyi	Cat# 130-045-201

**Deposited Data**		

RNA-seq files (UT, T, C, CT)	This Paper	GEO GSE129906

**Experimental Models: Cell Lines**		

THP-1	ATCC	TIB-202; RRID: CVCL 0006
Caco2	ATCC	HTB-37; RRID: CVCL 0025

**Oligonucleotides**		

Primer specific for *OSM*: Forward > cttccccagtgag gagacc	Roche	N/A
Primer specific for *OSM*: Reverse > ctgctctaagtcg gccagtc	Roche	N/A
Primer specific for *HBEGF*: Forward > tggggcttctc atgtttagg	Roche	N/A
Primer specific for *HBEGF*: Reverse > catgcccaac ttcactttctc	Roche	N/A
Primer specific for *GAPDH*: Forward > ccccggtttc tataaattgagc	Roche	N/A
Primer specific for *GAPDH*: Reverse > cttccccatgg tgtctgag	Roche	N/A

**Software and Algorithms**		

Bioinformatics & Evolutionary Genomics	Ghent University	http://bioinformatics.psb.ugent.be/webtools/Venn/
FlowJo 10	Tree Star	https://www.flowjo.com
GSEA version 3.0	Subramanian et al., 2005	https://software.broadinstitute.org/gsea/index.jsp
Heatmapper	Wishart group, University of Alberta	http://www.heatmapper.ca
ImageJ Version 1.8	NIH	https://imagej.nih.gov/ij/index.html
Partek Flow	Partek	http://www.partek.com/partek-flow/
Prism Version 6.0b	Graphpad	https://www.graphpad.com
ZellExplorer	Zellkraftwerk GmbH	http://www.zellkraftwerk.com/products/

**Other**		

5-OP-RU-MR1-Tetramer PE	NIH tetramer core facility	N/A
PerColl	GE Healthcare (mft)/ Merck	Cat# GE17-0891-01
Human *IL26* TaqMan Probe	Thermo Fisher Scientific	Hs00218189_m1
TaqMan Fast Advanced Master Mix	Thermo Fisher Scientific	Cat# 4444557
Zellsafe Tissue chips	Zellkraftwerk GmbH	no. 28050606/02-010

### Lead Contact and Materials Availability

Further information and requests for resources and reagents should be directed to and will be fulfilled by the Lead Contact, Paul Klenerman (paul.klenerman@medawar.ox.ac.uk). Please note that this study did not generate new unique reagents.

### Experimental Model and Subject Details

#### Human samples

All tissue samples were collected with appropriate patient consent and NHS REC provided ethical approval (reference numbers 09/H0606/5 for IBD patients and 16/YH/0247 for CRC patients and polyp biopsies).

Healthy PBMCs were isolated from leukocyte cones (NHS Blood Services). For long-term storage, PBMCs were kept in liquid nitrogen with freezing media (10% DMSO, 90% fetal calf serum, both Sigma-Aldrich). These samples are fully anonymized, so data on age and gender are not available for comparison. Colonic tissues were collected in the form of polyp biopsies or from the uninvolved mucosa of patients with colorectal cancer. Patient information is shown in the table below. All patients involved gave written consent. Colonic tissues were digested at 37°C for overnight with Collagenase A (Roche) and DNase I (Sigma-Aldrich). Colonic lymphocytes were then isolated from the cell suspension by a Percoll- (GE Healthcare) gradient: cells were resuspended in 4ml of a 40% Percoll solution that was carefully overlayed over 4ml of 80% Percoll. After centrifugation (2000rpm, 20min, brake turned off), lymphocytes were obtained from the interphase between the two Percoll layers. A detailed protocol has been described by Geremia et al. (2011).

Characteristics of the CRC patients.

**Table:** 

CRC patients	n = 21
Age (Average, SD∗)	68, 12
Sex (Male/Female)	12/9
Uninflamed, tumor-free tissue origin	
Small intestine	0
Large intestine	21
Time since diagnosis (years)	1, 1
Average, SD	

∗ SD: standard deviation

#### Cell lines

Cell lines were cultured at 37°C in 5% CO_2_. Caco2 cells (Colorectal adenocarcinoma cell line, ATCC) were cultured at a starting density of 4x10^5^ cell/cm^2^ in T-175 cell-culture flasks, using GlutaMAX medium supplemented with 10% fetal bovine serum (FBS), 1% MEM Non-essential-amino acid solution (NEAA), 100ug/mL Penicillin-Streptomycin, 2mM L-glutamine (Sigma Aldrich). Cultures were maintained with media exchange every second day and routinely split every week when cells had reached approximately 70% confluency.

THP1 cells (Human monocyte cell line, ATCC) were cultured at a density between 2x10^5^ to 10^6^ cells/mL in T-175 cell-culture using RPMI-1640 medium (Sigma Aldrich) supplemented with FBS, Penicillin-Streptomycin and L-glutamine.

### Method Details

#### Isolation and short-term culture of human lymphocytes

PBMCs were thawed, washed and maintained in RPMI 1640 with 10% fetal calf serum, 1% L-glutamine, and 1% penicillin/streptomycin (R10) (all Sigma-Aldrich). CD8^+^ T cells were positively labeled with CD8 Microbeads (Miltenyi Biotech, purities were ≥ 90%), and enriched from PBMCs using MS or LS columns following the manufacturer’s instructions (Miltenyi Biotech). Colonic lymphocytes were used without prior enrichment by CD8 microbeads and were maintained in R10 supplemented with 25ng/mL amphotericin B (GIBCO), 40 μg/mL gentamicin (GIBCO), and 10 μg/mL ciprofloxacin (Sigma-Aldrich).

#### *In vitro* stimulations

For non-specific TCR triggering, PBMCs, enriched CD8 T cells, sorted cells or colonic lymphocytes were stimulated with plate bound anti-CD3/28 antibodies (Miltenyi), or anti-CD3/28 beads (Miltenyi) at 1:1 ratio. ELISA plates (Greiner) were coated with 5 μg/mL anti-CD3/28 with the final volume of 100 μL at 4°C for overnight. Antibody mix was washed off the next morning, and plates were used after 1-hour 37°C incubation with R10. Anti-CD3/28 beads were prepared following the manufacturer’s instructions.

MAIT-specific TCR triggering was achieved by co-culturing of 2x10^5^ MACS-enriched CD8s with 1x10^5^ THP1 cells which had been previously pulsed with 10nM 5-OP-RU (kindly provided by David Fairlie) for 2 hours. Unpulsed THP1s were used as controls.

For cytokine triggering, cells were stimulated for 20 hours with IL-12 (Miltenyi) at 2ng/mL, IL-18 (MBL) at 50ng/ml, IL-15 (Miltenyi) at 25ng/ml, TL1A (R&D) at 100ng/ml, unless otherwise stated.

For activation of MAIT cells by bacteria-derived ligands, THP1 cells were loaded with PFA-fixed (2%, 20min) *E. coli* (DH5α, Invitrogen) at a 25 bacteria per cell (BpC) ratio overnight. Bacterially loaded THP1s were washed and co-cultured with MACS-enriched CD8s at a 1:2 ratio. In order to block the TCR-dependent component of this activation, in some experiments an anti-MR1 blocking antibody (26.5, Biolegend) was added to the co-cultures.

#### Flow cytometry

Brefeldin A (eBioscience, 1000x) was added into the cell cultures for the last 4 hours before intracellular staining.

Cells were stained with the antibodies and dyes listed in the Key Resources Table and were fixed with 2% Paraformaldehyde for 10 min before acquisition on a MACSQuant cytometer (Miltenyi) or LSRII (BD Biosciences). Data were analyzed with FlowJo (Tree Star Inc.), a representative gating strategy is shown in Figure S3A.

#### RNA sequencing (RNaseq)

CD8^+^ T cells were enriched from PBMCs of three healthy individuals and were rested overnight prior to sorting. On the next day, MAIT (CD161^2+^ Vα7.2^+^) cells were sorted using a Beckman Coulter MoFlo XDP and stimulated with a range of conditions including anti-CD3/28, cytokines (IL-12/IL-18/IL-15/TL1A), or the combination of both, or left untreated in R10 media for 24 hours. RNA was then extracted from these 12 samples using an RNeasy Micro kit (QIAGEN). The quantity and quality of extracted RNA was first evaluated using both a nanodrop spectrophotometer and the Agilent 2100 bioanalyzer. All samples had RNA integrity (RIN) values greater than 9 and were free from contaminating protein and organic compounds. RNaseq was performed by Wellcome Trust Centre for Human Genetics (University of Oxford) on a HiSeq4000v platform. Gene lists that were differentially expressed (> 4 fold, p < 0.01, FDR < 0.05) between various conditions and their normalized expression values, as well as the principle component analysis (PCA) plots, were generated with Partek® Flow®, an online analysis platform for Next Generation Sequencing data (http://www.partek.com/partek-flow/), following the user’s guide. Volcano plots were generated with Prism software. Heatmaps were generated using normalized counts with Heatmapper (http://www.heatmapper.ca/expression/) with the averaged linkage clustering method and Pearson distance measurement method. Venn diagrams were drawn with an online diagram drawing platform developed by Ghent University, Belgium (http://bioinformatics.psb.ugent.be/webtools/Venn/). Gene set enrichment analysis (GSEA) was performed using GSEA version 3.0 (Subramanian et al., 2005), comparing gene expression data as a whole with the reference gene list obtained from the publication by Linehan et al. (2018).

#### qPCR

CD161^2+^ Vα7.2^+^ (MAIT) and CD161^-^ Vα7.2^-^ cells were sorted from pre-enriched blood CD8^+^ T cells. These cells were then stimulated with anti-CD3/28, cytokines (IL-12/IL-18/IL-15/TL1A), the combination of both or left untreated in R10 media for 20 hours. The total RNA of sorted T cells was extracted with an RNeasy Micro kit (QIAGEN) and reverse transcribed using reverse transcribed using SuperScript III Reverse Transcriptase (Invitrogen). For detection of IL-26 mRNA, a 20X IL-26 human TaqMan® probe was used (Hs00218189_m1) with 2X TaqMan® Fast Advanced Master Mix (both from ThermoFisher Scientific). OSM and HEBGF cDNA quantification was performed with Roche ® hydrolysis probes (*OSM*: forward primer sequence, 5′-cttccccagtgaggagacc-3′, reverse sequence, 5′-ctgctctaagtcggccagtc-3′; HBEGF: forward primer sequence, 5′-tggggcttctcatgtttagg-3′, reverse sequence, 5′-catgcccaacttcactttctc-3′), with GAPDH as the internal control (forward primer sequence, 5′-ccccggtttctataaattgagc-3′, reverse sequence, 5′-cttccccatggtgtctgag-3′).

#### *In vitro* wound-healing assay

Enriched CD8^+^ were co-cultured with THP1 cells loaded with fixed *E. coli* at 25 BpC in the presence or absence of 20 μg/mL LEAF anti-MR1 Antibody (Biolegend). Supernatants were collected at 72 hours. A total of 1.5x10^4^ Caco2 cells were seeded per well in a 96-well clear flat bottom plate (Corning) and grown to confluency at 37°C for 5 days with media exchange every 2 days. Monolayers were scratched using a WoundMaker (Essen Bioscience), washed with serum-free medium and incubated with CD8^+^ 72h hour supernatants diluted 1:4 with fresh media. As a negative control, fresh media was used. Time lapse imaging was recorded every 4 hours using IncuCyte S3 Live Cell Analysis System (Essen Bioscience) for 36 hours at 37°C. GlutaMAX medium supplemented with 10% FBS, 1% NEAA, Penicillin-Streptomycin and L-glutamine was used throughout this experiment.

#### Chip Cytometry

Samples were snap frozen and cryosectioned onto cytometer chips (Zellsafe Tissue chips, Zellkraftwerk, GmbH, Deutscher Platz 5c, 04103 Leipzig, Germany). Sections were fixed *in situ* at room temperature for 10 minutes using 4% paraformaldehyde solution, then washed with 10-20 mL of PBS. Non-specific binding was blocked by incubating in 5% normal goat serum (Thermo Fisher cat016201) in PBS for at least one hour at room temperature. All antibodies were directly conjugated to their fluorophore and were diluted for the staining step in PBS. Immunostaining was performed using an iterative approach where up to three colors could be applied simultaneously. Fluorophores were subsequently bleached and a new round of antibodies applied to build up the panel (Hennig et al., 2009). Images were acquired using a Zellscanner One Chip cytometer (Zellkraftwerk) using the dedicated ZellExplorer software.

##### Data Integration

A selection of ImmGen (https://www.immgen.org/) samples (Heng et al., 2008) (microarrays) were merged first with a selection of RNA-Seq data from Hinks et al. (2019) and Linehan et al. (2018) (see also Fergusson et al., 2014). Raw read counts data were transformed to log2-counts per million (logCPM) using *voom* function in *limma* R package (Ritchie et al., 2015). RNA-Seq from Human MAIT cells were merged to mouse dataset using a common set of genes, using homologous ids from MGI database (http://www.informatics.jax.org/orthology.shtml). Batch effects were removed using *ComBat* function in ‘sva’ R package (Leek et al., 2019), using a procedure described in Johnson et al. (2007). Hierarchical clustering analysis of integrated expression profiles was performed using Euclidian distance as similarity measure and filtering genes by variance (IQR > 0.95).

### Quantification and Statistical Analysis

All graphs and statistical analyses, except RNaseq data analysis, were performed using GraphPad Prism Software Version 6.0b (La Jolla, CA). Statistical significance was assessed using paired Student’s t test, or repeated-measures two-way analysis of variances, with Bonferroni’s correction for multiple comparison assays. For the analysis of the RNaseq dataset, Partek Flow was used. For the *in vitro* wound-healing assay, ImageJ v1.8 was used to determine the area of wounds. Area at different time points were normalized as a percentage of the initial area. All data were presented as means ± SEM.

### Data and Code Availability

The accession number for the raw and pre-processed data from the RNaseq datasets reported in this paper is GEO: GSE129906.

## Consortia

The members of Oxford IBD Investigators are Carolina Arancibia-Carcamo, Adam Bailey, Eillie Barnes, Beth Bird-Lieberman, Oliver Brain, Barbara Braden, Jane Collier, James East, Alessandra Geremia, Lucy Howarth, Satish Keshav, Paul Klenerman, Simon Leedham, Rebecca Palmer, Fiona Powrie, Astor Rodrigues, Alison Simmons, Peter B. Sullivan, Simon P.L. Travis, and Holm H. Uhlig.

## Supplemental Information

Document S1. Figures S1–S6

Table S1. Transcriptional Alterations in MAIT Cells in Response to TCR, C, or Combined (TC) Stimulations, Related to Figure 4 Sheet 1 lists all the genes whose expression was significantly altered by the different stimulations compared to untreated (UT) MAIT cells. For each gene, p value, false discovery rate (FDR), and fold change are listed. Sheet 2 contains a list of 89 genes, whose expression changed, regardless of which stimulation as used.

Table S2. Comparison of Gene Expression among Stimulatory Conditions, Related to Figure 4 List of the p values, FDRs and fold changes compared to UT of each of the 1,594 genes whose expression was altered in at least one condition. Also contains gene expression values for each of the individual samples analyzed.

Table S3. Tissue-Repair Genes, Related to Figures 4 and 5 List of the tissue repair genes used by Linehan et al. (2018). Genes that showed altered expression in this study are marked in blue.

Document S2. Article plus Supplemental Information
