# Supplementary material for: TCR and Inflammatory Signals Tune Human MAIT Cells to Exert Specific Tissue Repair and Effector Functions
Source: Cell Rep. 2019 Sep 17;28(12):3077–3091.e5. doi: 10.1016/j.celrep.2019.08.050 (PMC6899450; doi:10.1016/j.celrep.2019.08.050)
Supplement: Document S1. Figures S1–S6 [file mmc1.pdf]

Cell Reports, Volume *28*

Supplemental Information

TCR and Inflammatory Signals Tune Human MAIT Cells to Exert Specific Tissue Repair and Effector Functions

Tianqi Leng, Hossain Delowar Akther, Carl-Philipp Hackstein, Kate Powell, Thomas King, Matthias Friedrich, Zoe Christoforidou, Sarah McCuaig, Mastura Neyazi, Carolina V. Arancibia-Cárcamo, Joachim Hagel, Fiona Powrie, Oxford IBD Investigators, Raphael Sanches Peres, Val Millar, Daniel Ebner, Rajesh Lamichhane, James Ussher, Timothy S.C. Hinks, Emanuele Marchi, Chris Willberg, and Paul Klenerman

Supplementary Figure 1

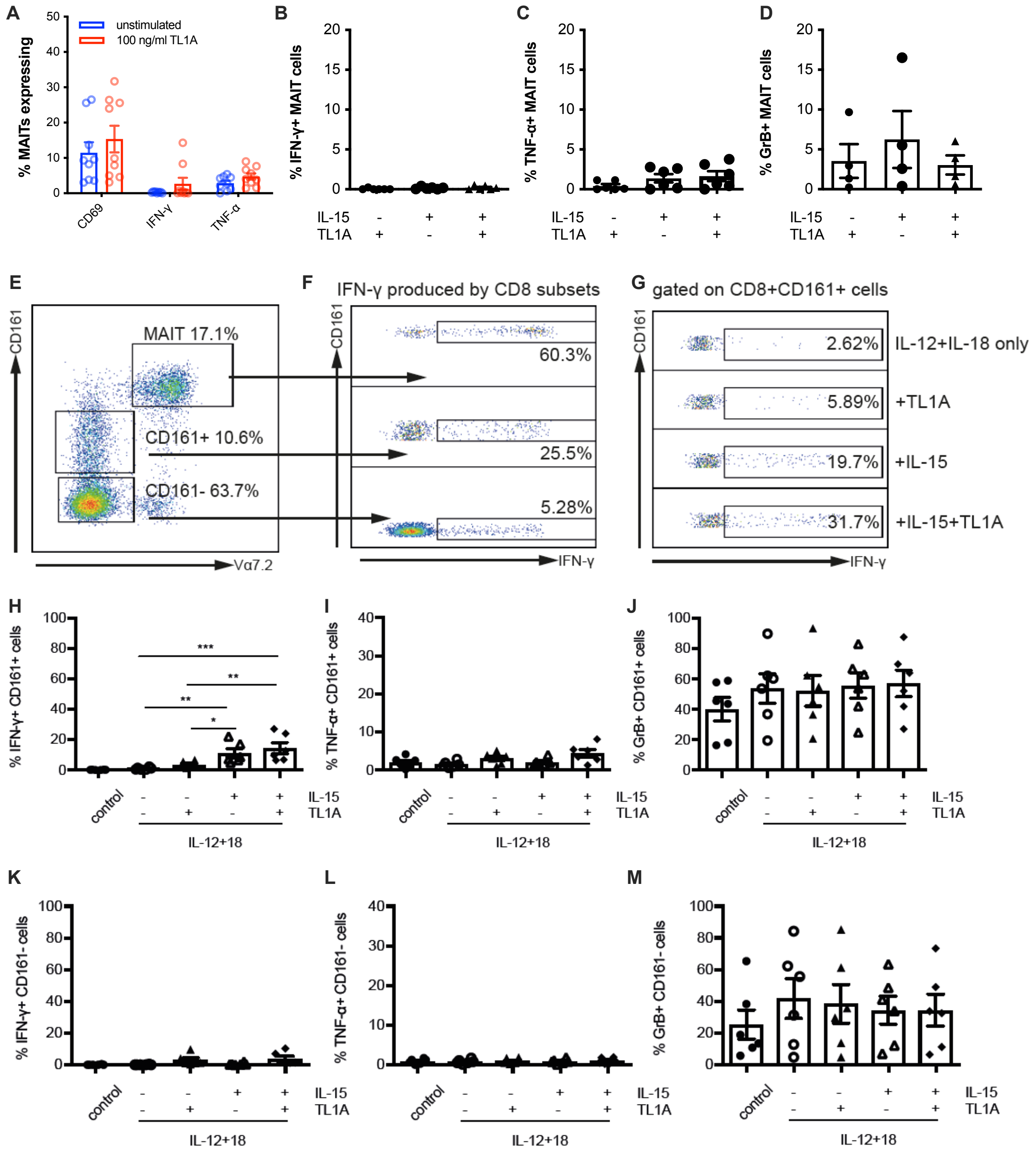

SFigure 1 (related to Fig 1). **TL1A and IL-15 alone do not promote MAIT cell effector functions and have only a limited effect on CD161+ and CD161- CD8+ T-cells.** CD8+ T cells were enriched from healthy PBMCs and stimulated overnight with combinations of the indicated cytokines. **(A)** Proportion of CD8+ MAIT cells producing CD69, IFN-γ, and TNF-αwhen left untreated, or stimulated singly with 100ng/ml TL1A. **(B-D)** Frequency of MAIT cells expressing IFN-γ (B), TNF-α (C) or GrB (D) upon stimulation with TL1A (100ng/ml), IL- 15 (25ng/ml) or both cytokines. **(E)** Gating strategy for CD8+ MAIT (CD161++Vα7.2+)/CD161+Vα7.2-/CD161-Vα7.2-cells **(F)** Proportion of CD8+MAIT (CD161++Vα7.2+), CD161+Vα7.2-, or CD161-Vα7.2-cells producing IFN-γ when stimulated with IL-12, IL-15, IL-18 and TL1A. **(G)** Proportion of CD8+CD161+Vα7.2-cells producing IFN-γ when stimulated with a range of conditions. **(H-M)** Proportion of CD8+CD161+Vα7.2- cells (H-J) or CD8+CD161-Vα7.2- cells (K-M) producing IFN-γ (H and K), TNF-α (I and L), or GrB (J and M) when treated with combinations of TL1A (100ng/ml) and IL-15 (25ng/ml) with suboptimal IL-12/18 (2ng/ml). Data were acquired from 6-8 donors in 2-3 experiments. Error bars represents mean ± SEM. Differences between the conditions were analysed by Friedman tests with Dunn's multiple comparison tests.

Supplementary Figure 2

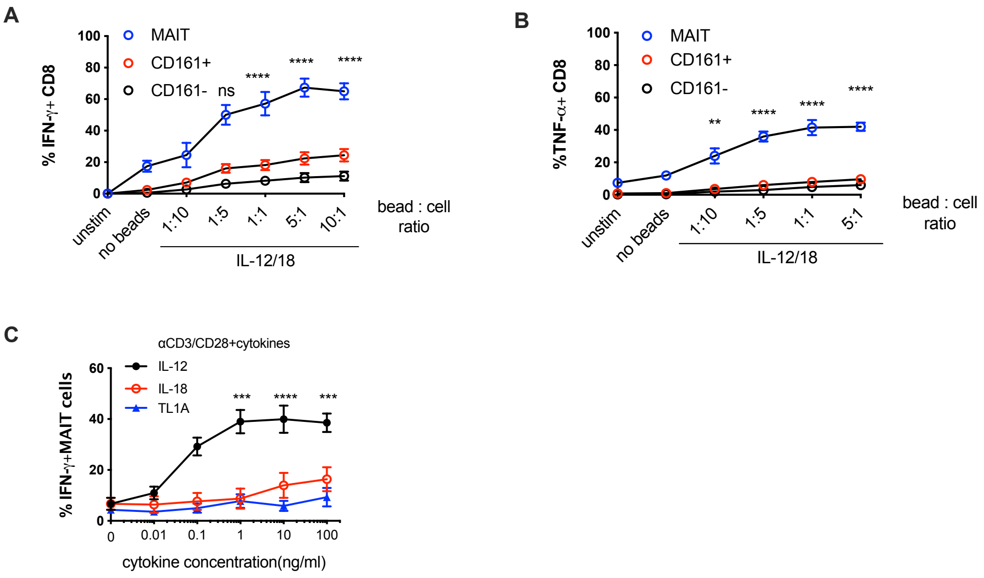

SFigure 2 (related to Fig. 2). **Functional studies on the impact of combined TCR and cytokine signalling.** CD8+ T cells were enriched from healthy PBMCs and stimulated in different ways. **(A, B)** Proportion of CD8+ MAIT (CD161++Vα7.2+)/CD161+/CD161- cells producing IFN-γ (A) or TNF-α (B) following overnight incubation with suboptimal concentrations of IL-12 and IL-18, plus αCD3/CD28 beads at increasing bead-to-cell ratios. **(C)** Proportion of CD8+MAIT cells producing IFN-γ (following stimulation with increasing concentrations of cytokines: IL-12, IL-18, or TL1A, respectively in the presence of plate-bound αCD3/CD28 antibodies. Data were acquired from 7-8 donors in three experiments. Differences between the conditions were analysed by 2way ANOVA with Tuckeys multiple comparison tests (A-C). Error bars represents mean ± SEM. ns = not significant, **$p<0.01$, ***$p<0.001$, ****$p<0.0001$.

Supplementary Figure 3

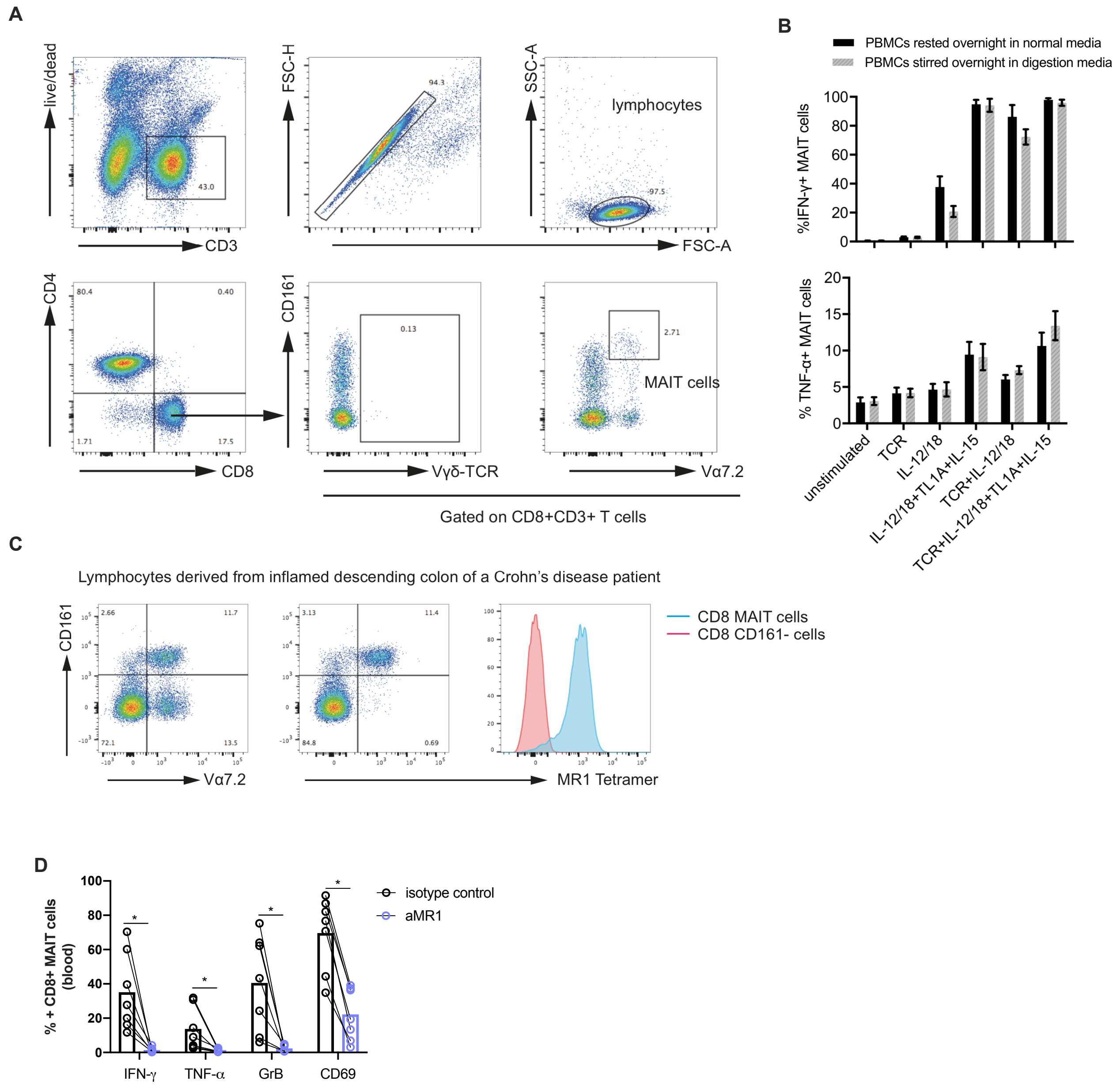

SFigure 3 (related to Fig. 3). **Identification of MAIT cells in the colonic lamina propria and additional functional studies on blood-derived MAITs. (A)** Gating strategy to identify CD8+MAIT cells from gut LPLs. **(B)** Proportion of CD8+MAIT cells producing IFN-γ or TNF-α after overnight stimulations. CD8+MAIT cells were derived from PBMCs, which, prior to stimulation, were either rested in the normal media or stirred in the digestion media containing DNase and Collagenase A for 12 hours. (**C**) Representative plot showing how to identify MAIT cells from the gut by using either a conventional Vα7.2 TCR staining antibody or the MR1-tetramer staining antibody, in combination with CD161 staining. **(D)** Proportion of CD8+ MAIT cell expressing the indicated molecules after overnight co-culture with THP1 cells incubated with 25 fixed *E. coli* bacteria per cell in the presence of a blocking antibody directed against MR1 or an isotype control. Data were acquired from 1-7 donors in 1-3 experiments. Differences between the conditions were analysed by Wilcoxon tests (D). *$p<0.05$

Supplementary Figure 4

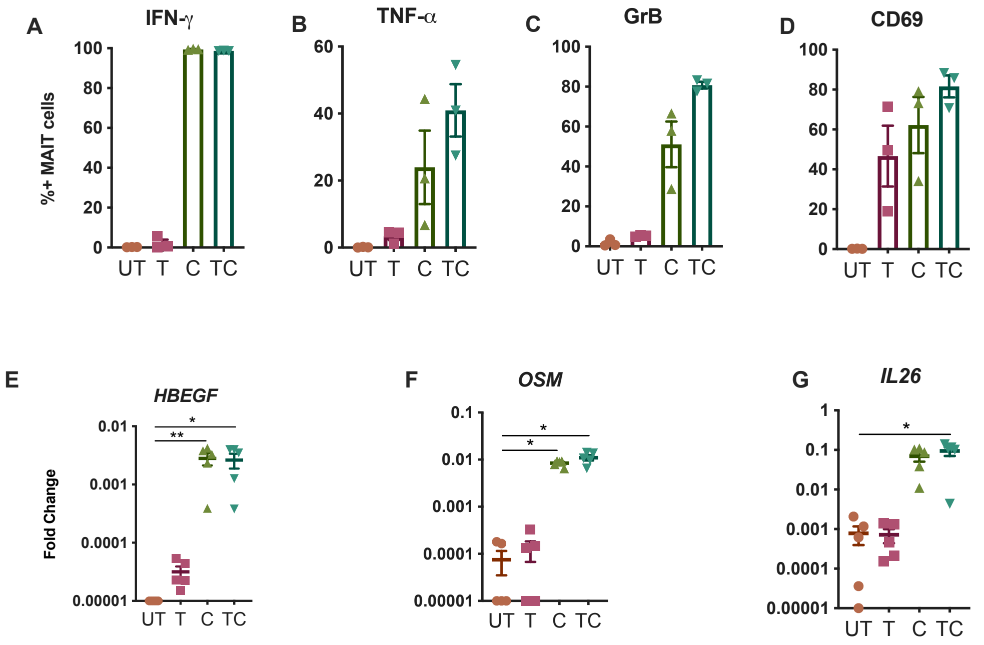

SFigure 4 (related to Fig. 4). **Expression of effector molecules by MAITs treated with the conditions used in the RNAseq study.** CD8+ T-cells were MACS enriched and left untreated (UT) or were stimulated with αCD3/28 beads (T), suboptimal IL-12/18 in combination with TL1A and IL-15 (C) or with a combination of the aforementioned cytokines and αCD3/28 beads (TC) overnight. **(A-D)** Proportion of CD8+MAIT cells isolated from parts of the samples used for the RNAseq experiment producing IFN-γ (A), TNF-α (B), GrB (C) or CD69 (D). Each dot corresponds to a donor of the RNAseq study, data were acquired from 3 donors in one experiment. **(E-G)** Expression levels of *HBEGF* (E), *OSM* (F) and *IL26* (G) in CD8+MAIT cells (n=5) examined by qPCR. GAPDH was used as house-keeping gene. Data were acquired from five donors in two experiments. Error bars represents mean ± SEM. Differences between conditions were analysed by Friedman tests with Dunn's multiple comparisons tests. *p<0.05, **p<0.01.

Supplementary Figure 5

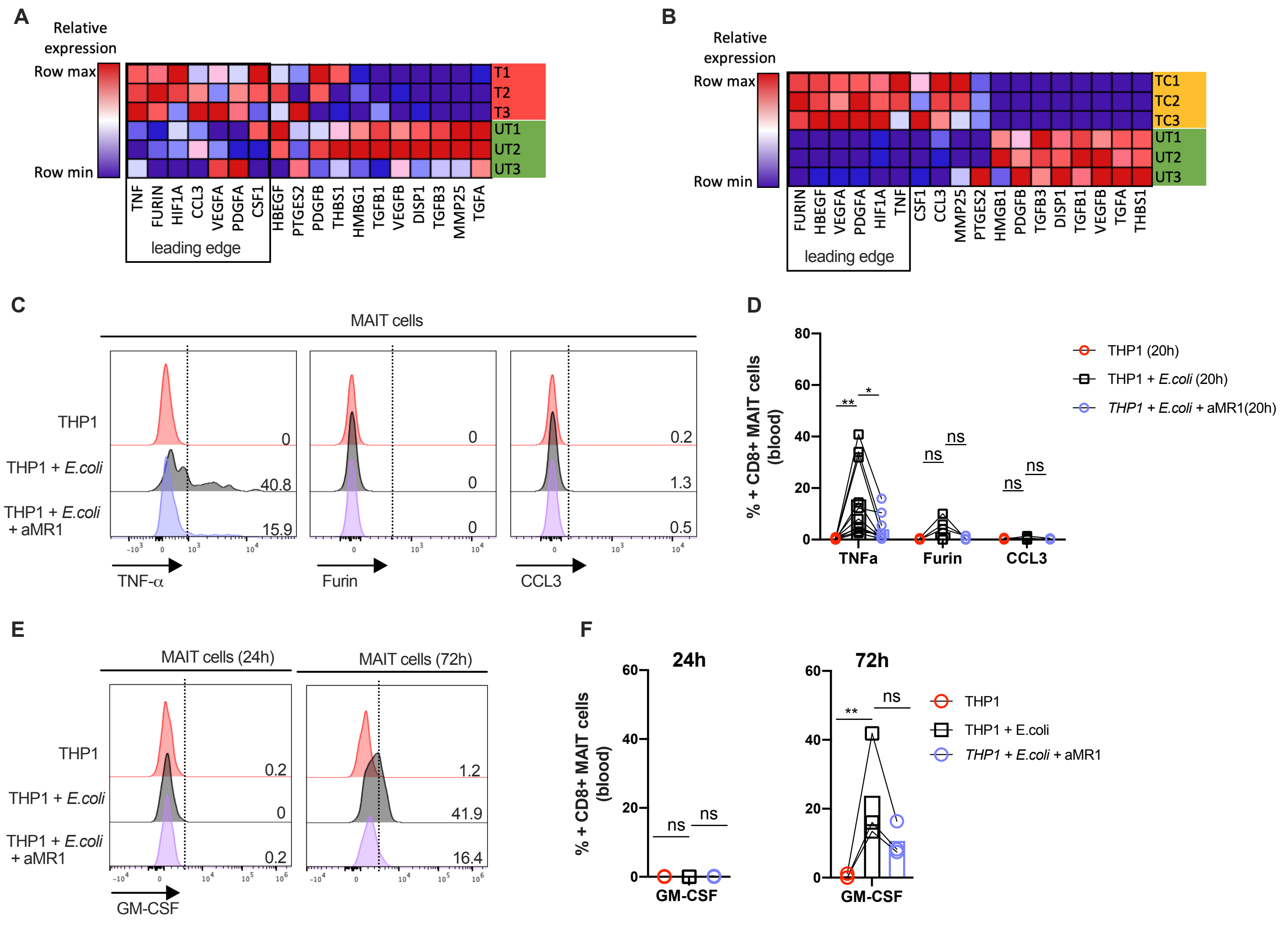

SFigure 5 (related to Fig. 5). **Further investigation of tissue repair related functions of MAIT cells. (A, B)** Relative expression of the genes of the tissue repair gene set by MAIT cells stimulated by TCR (A) or TCR+cytokines (B) compared to unstimulated controls. The leading edge genes of the corresponding GSEA plots (F6) are marked. The original tissue repair gene set of 101 genes was restricted to the genes present in our dataset. Data were acquired from 3 healthy donors in one experiment. **(C)** Flow cytometry analysis of the expression of TNF-α, Furin and CCL3 by CD161++/MAIT CD8+ T cells in response to fixed *E. coli* presented by THP1 cells in the presence or absence of an anti-MR1 (αMR1) blocking antibody at 20h timepoint. (**D**) Statistical analysis of the expression of the effector molecules shown in (A). Data were acquired from seven donors in three experiments. **(E)** Flow cytometry and **(F)** statistical analysis of the expression of GM-CSF by CD161++/MAIT CD8+ T cells at 20h and 72h timepoints using the conditions described in (A). Data were acquired from three donors in one experiment.

Supplementary Figure 6

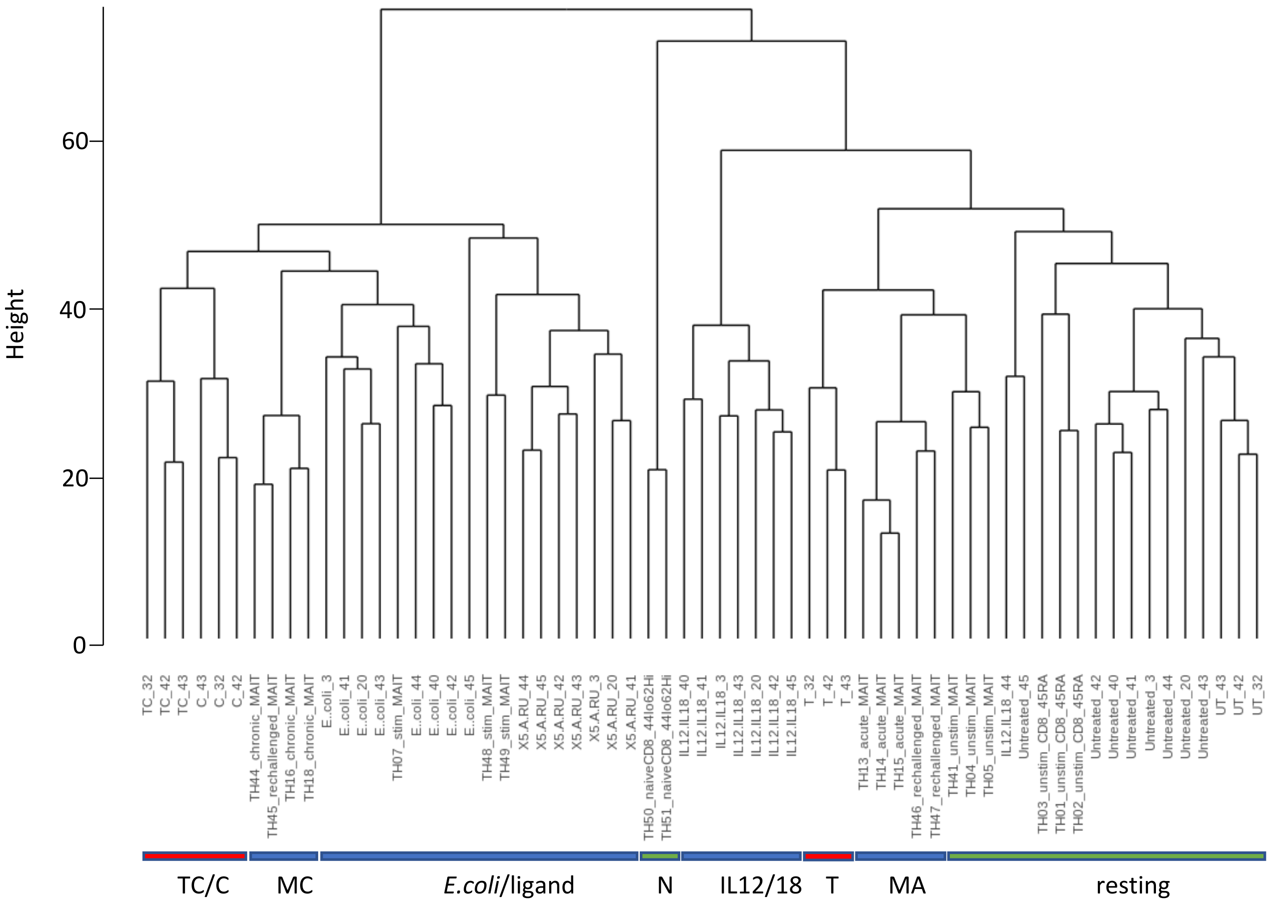

SFigure 6 (related to Fig. 6). **Data fusion of three recently generated human and mouse MAIT datasets.** In this plot we have extended the data fusion shown in Figure 6, by including the human datasets from Lamichhane et al (Lamichhane et al., 2019). The other background data have been removed. Cells are indicated as either naïve or unstimulated (N), acutely activated in vivo (MA) or chronically activated in vivo (MC). Similarity between the expression profiles is measured using a Euclidean distance (Height).
